# Updated benchmarking of variant effect predictors using deep mutational scanning

**DOI:** 10.15252/msb.202211474

**Published:** 2023-06-13

**Authors:** Benjamin J Livesey, Joseph A Marsh

**Affiliations:** ^1^ MRC Human Genetics Unit, Institute of Genetics and Cancer University of Edinburgh Edinburgh UK

**Keywords:** Benchmark, Circularity, DMS, MAVE, VEP, Computational Biology, Genetics, Gene Therapy & Genetic Disease

## Abstract

The assessment of variant effect predictor (VEP) performance is fraught with biases introduced by benchmarking against clinical observations. In this study, building on our previous work, we use independently generated measurements of protein function from deep mutational scanning (DMS) experiments for 26 human proteins to benchmark 55 different VEPs, while introducing minimal data circularity. Many top‐performing VEPs are unsupervised methods including EVE, DeepSequence and ESM‐1v, a protein language model that ranked first overall. However, the strong performance of recent supervised VEPs, in particular VARITY, shows that developers are taking data circularity and bias issues seriously. We also assess the performance of DMS and unsupervised VEPs for discriminating between known pathogenic and putatively benign missense variants. Our findings are mixed, demonstrating that some DMS datasets perform exceptionally at variant classification, while others are poor. Notably, we observe a striking correlation between VEP agreement with DMS data and performance in identifying clinically relevant variants, strongly supporting the validity of our rankings and the utility of DMS for independent benchmarking.

## Graphical abstract

Common sources of bias in variant effect predictor benchmarking are assessed using data from deep mutational scanning experiments. ESM‐1v, EVE and DeepSequence are among the top performers on both functionally validated and clinically observed variants.

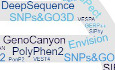

## Introduction

Accurately classifying variants of uncertain clinical significance remains an ongoing challenge for variant interpretation. Single‐nucleotide variants are the most common type of genetic variation in humans, most of which have no role in disease (Auton *et al*, 2015). Pathogenic variants are enriched among the rarest occurring variants in the human population (Wang *et al*, 2021), which makes gathering sufficient evidence to classify them a challenging prospect, while identification of rare benign variants is arguably an even greater challenge (Niroula & Vihinen, 2019). Over the past two decades, the field of computational variant effect prediction has sought to provide additional evidence for the classification of variants of uncertain significance often identified in genetic sequencing data (Livesey & Marsh, 2022). Variant effect predictors (VEPs) are algorithms that use evidence from various sources, including evolutionary conservation, functional annotations and physicochemical differences, to predict the likely phenotypic outcome of a genetic variant. The output of VEPs must be benchmarked against a “gold standard” to ensure that the predictor is generating accurate results (Sarkar *et al*, 2020). Such benchmarking studies are frequently conducted both by VEP authors and independent groups, traditionally by comparing VEP classifications to sets of known pathogenic and benign variants (Gunning *et al*, 2021). This approach raised some concern over the potential for data circularity (the reuse of data) to inflate VEP performance estimates (Grimm *et al*, 2015). Type 1 circularity involves recycling data originally used to train a predictor while assessing its performance, leading to improved performance compared with more appropriate benchmarking data. Type 2 circularity occurs when a VEP identifies a gene where mutations are highly skewed towards either a pathogenic or a benign outcome. In these cases, future predictions on mutations in this gene may be influenced by a VEPs previous experience, often resulting in apparent good performance in other mutations in these proteins, but much poorer performance on novel proteins or genes with mixed clinical outcomes associated with mutations.

We previously attempted to address the issue of data circularity by using data from deep mutational scanning (DMS) studies as the “gold standard” to perform a benchmark of VEP performance against single amino acid variants (Livesey & Marsh, 2020). DMS encompasses a wide variety of high‐throughput experimental techniques, whereby functional scores for large numbers of amino acid variants are measured (Fowler & Fields, 2014). Because most DMS‐derived functional scores are for variants never observed in the human population, using them to assess VEP performance can address the issues of limited benchmarking data availability that sometimes lead to type 1 circularity. Even DMS‐derived variants that exist in VEP training data have functional scores fully independent from previous clinical labels. Our study also used the correlation between the continuous outcome of each VEP and the DMS functional scores as the basis for our benchmark. This approach helps to address type 2 circularity as a VEP cannot score highly by assigning all variants in a protein as a single class but must determine the relative functional impact of each variant. Previously, we identified a method based on unsupervised machine learning, DeepSequence (Riesselman *et al*, 2018), to be the top‐performing VEP for human proteins. We also demonstrated the ability of DMS to outperform VEPs at direct classification of clinically relevant variants.

Significant progress has been made in both VEP development and DMS methodologies since our previous study with multiple predictors based on cutting‐edge machine learning techniques and many new DMS studies being published (preprint: Meier *et al*, 2021; Wu *et al*, 2021). In this paper, we have updated our previous benchmarking strategy with the addition of more recently published VEPs and many additional human DMS datasets. While benchmarking VEPs against DMS datasets greatly mitigates the issue of data circularity, the relevance of such datasets to human pathogenic conditions may be more circumspect. For example, in the case of the dominant‐negative effect, one would expect mutations with a mild effect on individual protein function to cause a more severe phenotype than highly destabilising mutations. Other factors such as limitations of the experimental system and relevance of the functional assay to disease mechanisms can also affect the usefulness of such datasets. To complement our analysis and help assess the usefulness of DMS for benchmarking, we have also assessed the performance of DMS datasets and unsupervised VEPs against known pathogenic and putatively benign missense variants. The remarkable correlation between VEP ranking using our two independent benchmarks provides strong support for our rankings and demonstrates the utility of using DMS data for the task of VEP assessment.

## Results

### Overview of VEPs and DMS datasets used in this study

Compared with our previous benchmark, we increased the number of DMS datasets of human single amino acid variants from 13 to 26. We considered exclusively human proteins, as only a subset of the VEPs we include in this analysis can generate predictions for nonhuman proteins. We identified new and previously unused DMS datasets through searching MaveDB (Esposito *et al*, 2019) and identifying recently published works in the literature. Table 1 summarises each of the new DMS studies that were added to the analysis, with the full set of DMS experiments given in Table EV1.

**Table 1:** Summary of new DMS studies used to benchmark VEPs.

DMS target (Uniprot ID)	Functional assay	Coverage of all amino acid substitutions (%)	Reference
***SNCA*** (P37840)	Yeast growth rate hindered by aggregate toxicity (reverse survival)	97.26	Newberry *et al* (2020)
***CASP3*** (P42574)	Apoptotic activity assessed by fluorescence in a microfluidic system.	28.63	Roychowdhury & Romero (2022)
***CASP7*** (P55310)		29.17	
***CBS*** (P35520)	Yeast growth rate complementation	64.41	Sun *et al* (2020)
***CCR5*** (P51681)	Antibody binding activity and surface expression levels in human cells	99.97	Heredia *et al* (2018)
***CXCR4*** (P61073)	Antibody binding activity and surface expression levels in human cells	99.36	Heredia *et al* (2018)
***CYP2C9*** (P11717)	Activity profiling (Click‐seq).	65.97	Amorosi *et al* (2021)
***GDI1*** (P31150)	Yeast growth rate complementation	51.40	Preprint: Silverstein *et al* (2021)
***HMGCR*** (P04035)	Yeast growth rate complementation	99.89	Jiang (2019)
***LDLRAP1*** (Q5SW96)	Yeast two‐hybrid binding assay	99.03	Jiang (2019)
***MSH2*** (P43246)	Rescue of MMR‐deficient HAP1 cells	94.38	Jia *et al* (2021)
***MTHFR*** (P42898)	Yeast growth rate complementation	99.85	Weile *et al* (2021)
***NUDT15*** (Q9NV35)	Drug resistance assay (growth rate).	94.16	Suiter *et al* (2020)
***TP53*** (P04637)^a^	reverse growth rate assay in human cells	39.37	Kotler *et al* (2018)
***PDE3A*** (Q14432)	DNMDP sensitivity in a glioblastoma cell line.	36.41	Garvie *et al* (2021)
***VKORC1*** (Q9BQB6)	Protein stability assessed by FACS (VAMP‐seq).	87.02	Chiasson *et al* (2020)

All DMS studies used to benchmark VEPs that were not present in our previous benchmark including a brief description of the functional assay used to assess variant fitness. Less than 40% coverage of amino acid substitutions in the protein indicates that study focussed on SNVs or a single protein domain rather than amino acid variants across the whole protein.

^a^ Our previous benchmark already included TP53, but we identified a further dataset published by another group.

Many DMS datasets provide multiple scores covering different experimental conditions and sometimes entirely different fitness assays of the same protein; mappings between the original names of these assays in their respective papers and MaveDB and identifiers used in this study are provided in Table EV2. We calculated the absolute Spearman's correlations between these score sets in the same protein to gauge the reproducibility of DMS results under different conditions. The strongest correlations (> 0.9) were between experiments in extremely similar conditions, while assays investigating fitness under highly varying experimental conditions or using alternate fitness metrics often resulted in much lower correlations (< 0.3). Most correlations observed between alternative assays were in a range between 0.4 and 0.6 (median 0.54; Table EV3), which is similar to the level of correlation between DMS and the top VEPs in our previous study. To represent each protein in our analysis, we selected a single assay from each DMS study. For proteins with multiple DMS datasets available, the assay that gave the highest median absolute Spearman's correlation against all VEPs was selected to be representative of fitness effects in each DMS target protein (Table EV1). The use of the median ensures that our assay selection is not skewed by a few particularly high‐ or low‐correlating VEPs.

We also added 12 new predictors to this study, bringing the total number of VEPs, conservation scores and substitution matrices benchmarked from 46 to 55 (accounting for a handful removed due to inaccessibility or suitability). Several of the new VEPs included in this analysis were added to the dbNSFP database in the 4.2 update (Liu *et al*, 2020), while others were identified by literature search. A summary of the new VEPs assessed in our benchmark along with their sources is provided in Table 2, while the full list of VEPs is available in Table EV4. We did not include any methods focussed on predicting the effects of variants on protein stability, but several of these have been assessed in a recent study (preprint: Gerasimavicius *et al*, 2023).

**Table 2:** All benchmarked VEPs that were not present in our previous study.

VEP	Classification	Data source	Reference
ESM‐1v	Unsupervised (no fine‐tuning)	Run locally	Preprint: Meier *et al* (2021)
LIST‐S2	Unsupervised	dbNSFP 4.2	Malhis *et al* (2020)
EVE	Unsupervised	Run locally and https://evemodel.org/download/bulk	Frazer *et al* (2021)
EVmutation_epistatic and EVmutation_independent	Unsupervised	Run locally	Hopf *et al* (2017)
VESPAl	Unsupervised	https://zenodo.org/record/5905863#.Yuu0Y3bMI2w	Marquet *et al* (2021)
mutationTCN	Unsupervised	http://mtban.kaist.ac.kr/humanProteins.jsp	Kim & Kim (2020)
ClinPred	Supervised	dbNSFP 4.2	Alirezaie *et al* (2018)
BayesDel	Supervised	dbNSFP 4.2	Feng (2017)
MetaRNN	Supervised	dbNSFP 4.2	Preprint: Li *et al* (2021)
VARITY_R and VARITY_ER	Supervised	http://varity.varianteffect.org/	Wu *et al* (2021)

The classification and data sources of all VEPs that are benchmarked in this study, but not included in our previous analysis. Note that ESM‐1v can be fine‐tuned to a protein of interest by providing a multiple sequence alignment; we used ESM‐1v in a zero‐shot context with no task‐specific training or fine‐tuning.

Previously, we defined four different categories to classify VEPs based on their architecture and training: supervised, unsupervised, empirical and metapredictors. These categories overlapped with each other to some extent as several VEPs could fall into multiple categories. To better reflect which predictors are related by methodology, we have now given all VEPs a label that is either “supervised” or “unsupervised” (Table 2; Table EV4), which reflects whether labelled examples were used to train the predictor and thus whether data circularity is a concern for its assessment. Despite this simplification of VEP classification, Eigen could still qualify for both categories. Eigen uses an unsupervised spectral method to combine multiple other VEP scores and deleteriousness metrics. However, one of the VEPs it includes as a feature is PolyPhen‐2, a supervised VEP that has been trained on labelled variants. Thus, Eigen has the potential for data circularity, and we have therefore labelled it as supervised in this analysis.

### Benchmarking of VEPs using DMS data

**Figure 1:**
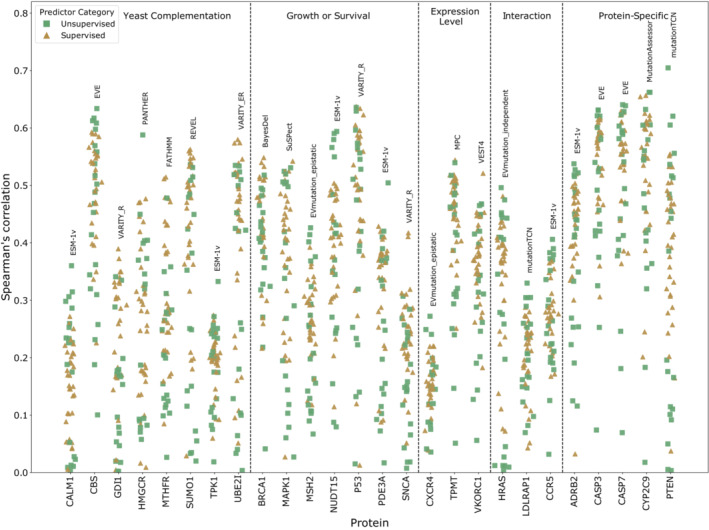
Spearman's correlation between DMS datasets and VEPs The Spearman's correlation between all VEPs and the selected DMS datasets for every protein. The top‐performing VEP by Spearman's correlation for each protein is labelled on the plot. DMS experiments are grouped by the type of fitness assay employed. “Yeast complementation” describes an assay where the human gene is used to compensate for the lack of activity in an essential yeast gene. “Growth or survival” includes any assay where growth rate (and lack of growth) is assessed excluding yeast complementation assays. “Expression level” includes VAMP‐seq and other assays that quantify the amount of protein produced. “Interaction” includes any assays that quantify a protein's interaction with binding partners such as yeast two‐hybrid. “Protein specific” includes any assay tailored to assessing the function of a particular protein that does not fall easily into another category.

**Figure EV1:**
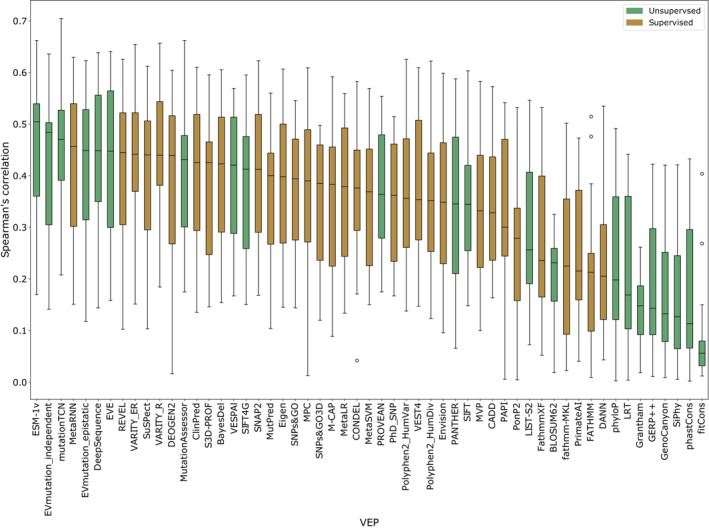
Distribution of Spearman's correlations between VEPs and DMS datasets The distribution of Spearman's correlations between all VEPs and each selected DMS dataset ranked by the median correlation (black bar). The boxplot whiskers indicate the range of the data while flier points are represented by empty circles.

We calculated the Spearman's correlation between each of the selected representative DMS datasets for every protein, and all available variant effect predictions using the continuous outcome scores produced by each VEP. Our results show that many of the recently developed VEPs produce higher correlations than those already present in our previous analysis (Fig 1). The correlations also varied considerably between each DMS dataset (Fig EV1). Of particular note are the unsupervised methods EVE and ESM‐1v as well as the supervised predictor VARITY. EVmutation is a slightly older unsupervised VEP that was not included in our previous study but also produced high correlations with the DMS data.

Low correlations with all VEPs were observed for several DMS datasets in our previous study, notably TPK1 and CALM1. The expansion of this analysis with further DMS datasets has highlighted additional cases where all VEPs fall below 0.4 Spearman's correlation with the DMS data: CXCR4, GDI1 and LDLRAP1. Interestingly, all but one of the DMS datasets were carried out in yeast systems (complementation assays in CALM1, TPK1, GDI1 and a two‐hybrid assay for LDLRAP1); the exception was CXCR4, which was assessed in human cells by expression level. On the contrary, some yeast assays did show high correlations, so it is likely that there are strong protein‐specific factors influencing this trend. Some of the highest correlations between VEP output and DMS results observed in this study involved DMS assays that were tailored specifically to the function of the protein being assessed (“Protein‐specific assays” in Fig 1). Other common DMS approaches such as measuring protein expression levels by VAMP‐seq (Matreyek *et al*, 2018), cell‐surface expression, or measuring specific protein interaction affinities tended to be less correlated with VEP predictions or produced mixed results. This is likely due to a disconnect between the specific fitness definition of the assay and the more general fitness effects predicted by VEPs. VAMP‐seq, for example only identifies variants that negatively affect protein stability as low fitness, while the protein itself may be nonfunctional but stable.

**Figure 2:**
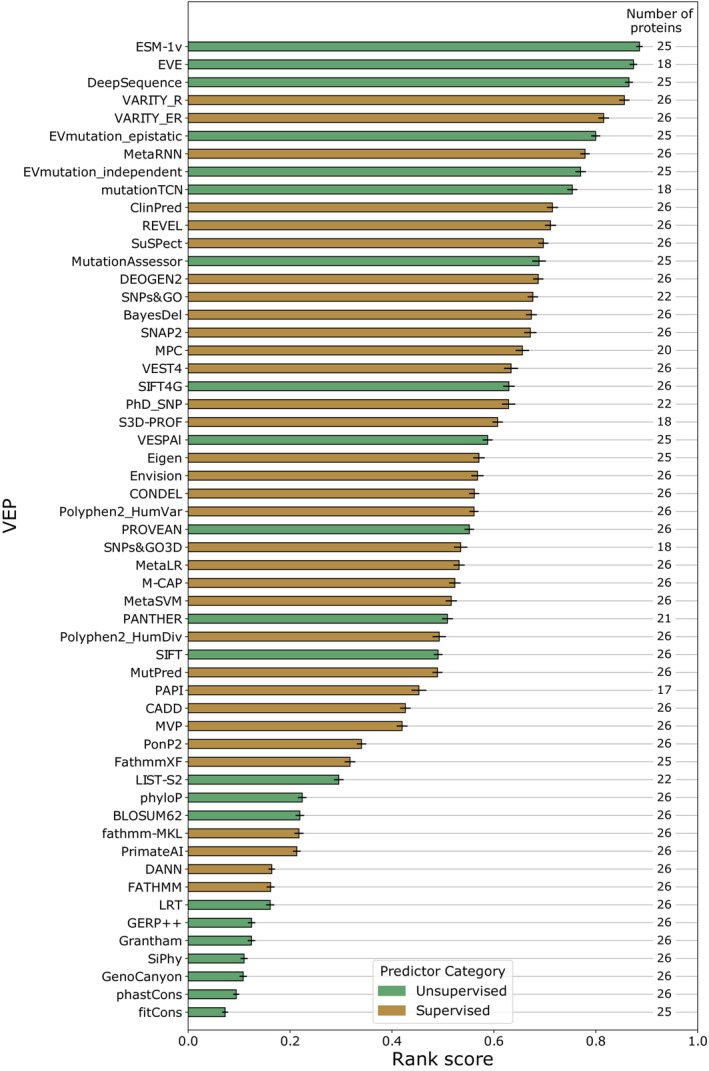
Overall ranking of VEP performance based on correlation with DMS data Rank scores for each VEP based on the Spearman's correlation between VEP predictions and DMS data across all proteins using only shared variants by pairwise comparisons. The number of proteins for which predictions of each VEP are available is indicated on the right of the plot. Error bars represent the standard deviation of rank scores obtained in 1000 bootstrap iterations.

We improved upon our previous VEP rank score calculations by performing a comparison between all pairs of VEPs using the Spearman's correlation between each VEP and DMS data across only variants for which both VEPs produced predictions. This resolves the issue of VEPs being compared across variants that are not necessarily shared between them. For example, some VEPs output predictions for every possible amino acid substitution, while others output predictions only for missense variants possible via a single‐nucleotide change. Moreover, some VEPs do not output predictions across the entire length of the protein. According to our methodology, VEPs receive a point for “winning” each pairwise comparison, and the total score is then divided by the number of comparisons the VEP participated in. We averaged this metric for each VEP across all DMS datasets to produce a final rank score that can be interpreted as the average proportion of other VEPs that each VEP performs better than across all DMS datasets (Fig 2). The per‐protein results are available in Table EV5.

To determine the significance of these rankings, we implemented a bootstrapping approach whereby all pairs of VEP scores were resampled with replacement and the rankings recalculated 1,000 times (Dataset EV1). Using both the new (Fig 2) and old (Table EV6) ranking methods, the top‐ranked VEP was ESM‐1v, a new unsupervised protein language model that produces functional predictions by zero‐shot inference (preprint: Meier *et al*, 2021), although its ranking did not differ significantly from EVE (*P* = 0.130). Like many other unsupervised predictors, ESM‐1v is trained using large numbers of protein sequences, but unlike other methods, ESM‐1v is not trained using an alignment specifically related to the protein of interest. It is instead pretrained on a large database of 98 million protein sequences. Zero‐shot prediction is the application of a model to an entirely new task without any task‐specific training (Lampert *et al*, 2009). While ESM‐1v can be fine‐tuned by providing a multiple sequence alignment (MSA), here the preconstructed model is used to directly infer fitness effects for any protein with no additional training for the target proteins or fine‐tuning. In addition to performing top in our analysis, ESM‐1v is considerably faster and easier to run than other top unsupervised methods (EVE, DeepSequence and EVmutation), as generating an MSA or training a new model for every protein is not required.

VARITY was the top‐ranking supervised VEP in our analysis, being significantly outperformed only by ESM‐1v (*P* = 0.006). VARITY is based on a gradient boosted tree model and has an innovative approach to weighting training data by predicted quality (Wu *et al*, 2021). The model gives two scores, VARITY_R which includes only rare pathogenic variants (minor allele frequency < 0.5%) in the core training set and VARITY_ER which includes only extremely rare pathogenic variants (minor allele frequency < 1 × 10^−6^). It must be noted that, like Envision (Gray *et al*, 2018), VARITY uses some DMS datasets during its training, specifically 10 of the same datasets we have used to assess predictors in this analysis (*UBE2I*, *SUMO1*, *CALM1*, *TPK1*, *GDI1*, *MTHFR*, *CBS*, *BRCA1*, *PTEN* and *TPMT*); therefore, data circularity may be inflating the performance estimates of VARITY. Importantly, however, after exclusion of these DMS datasets from the benchmarking analysis, VARITY_R and VARITY_ER retain 4^th^ and 5^th^ ranked places, respectively, and the rank scores even improve marginally (Dataset EV2). VARITY_R was also not significantly lower ranked than DeepSequence (*P* = 0.201) or ESM‐1v (*P* = 0.122) using this subset of DMS datasets, but EVE was significantly higher ranked than all other VEPs. Thus, the strong performance of VARITY does not appear to be due to data circularity, although the possibility also exists that VARITY has learned to predict some features of DMS data in general.

The other top performers, EVE (Frazer *et al*, 2021) and DeepSequence, were both developed by the same group, and each makes use of an unsupervised variational autoencoder to learn the latent rules underlying a multiple sequence alignment based on the protein of interest. Performance of the two VEPs is very similar, with EVE ranking slightly higher, although not significantly better than DeepSequence (*P* = 0.123) or VARITY_R (*P* = 0.062). EVE scores are constrained to a range between 0 and 1 to aid with interpretability and precalculated results are available to download online, while DeepSequence outputs unconstrained log‐likelihood ratios and do not offer any precalculated results.

One factor that affects the usefulness of VEPs is the proportion of mutations for which they can produce results. Some VEPs do not provide predictions in low‐coverage MSA regions by default. Other VEPs generate predictions only at the nucleotide level and thus have no output for amino acid substitutions that require multiple nucleotide changes. Our ranking system could potentially favour low‐coverage VEPs in cases where they fail to produce outputs in a generally poorly predicted region of a protein. To account for this possibility while not unfairly penalising nucleotide‐level VEPs, we considered only missense mutations possible via a single‐nucleotide change and, on the assumption that most missing data would be due to poorly conserved protein regions, we filled the remaining missing scores for each VEP with the most benign score it produced on a per‐protein basis. Recalculating the VEP rankings like this (Dataset EV3) does not greatly change the outcome, with ESM‐1v retaining its top position. EVE, which has the lowest coverage of the top VEPs (84.0%) drops from 2^nd^ to 4^th^ place, behind DeepSequence and VARITY_R. Most notably, mutationTCN (Kim & Kim, 2020), the VEP with the lowest coverage overall (64.5%), dropped from 9^th^ (significantly lower ranked than 8 other VEPs) to 20^th^ (significantly lower ranked than 19 other VEPs), indicating that some of its apparent performance may have been due to exclusion of poorly predicted regions.

The correlation of VEP predictions and DMS measurements varies along the length of a protein sequence with some regions being much more highly correlated than others. In regions of DMS maps that correlate poorly with all VEPs, comparison between VEPs is less meaningful; therefore, it may make sense to exclude these regions from the analysis. To address this issue, we used a scanning window of length 20 amino acids to calculate the average VEP correlation with DMS across each protein. We then removed the central 10 amino acids of any window that fell more than one standard deviation below the mean correlation across all windows. The remaining data were used to recalculate the rank scores (Dataset EV4). Only minor changes to the ranking of individual predictors were observed and all broad trends remained, EVE ranked slightly higher than ESM‐1v although the difference was not significant (*P* = 0.801).

### Performance of DMS compared with VEPs against datasets of pathogenic and benign missense variants

One of the most interesting applications of DMS data is in directly predicting the effects of clinically relevant variants. While data circularity often negatively influences our ability to evaluate supervised VEPs, known clinical labels have no impact on the assessment of experimentally derived, fully independent DMS data and, theoretically, a minimal impact on unsupervised VEPs. To assess the performance of DMS datasets at predicting actual clinical outcomes in comparison with unsupervised VEPs, we used known pathogenic and likely pathogenic missense variants from ClinVar (Landrum *et al*, 2014) and the Human Gene Mutation Database (HGMD) public (Stenson *et al*, 2003), while putatively benign variants were obtained from gnomAD (Karczewski *et al*, 2020), excluding those also in the pathogenic set. We refer to the gnomAD variants as “putatively benign” because the individuals sequenced are from cohorts without severe paediatric disease as well as their first‐ and second‐degree relatives. While gnomAD certainly contains some recessive, low‐penetrance and late‐onset pathogenic variants, it should be highly enriched in benign variants and provides a useful set for comparison with the known pathogenic variants from ClinVar and HGMD. In principle, the quality of gnomAD as a benign reference set could be improved by filtering out variants with low allele frequency. However, doing so drastically reduces the size of the benign datasets, resulting in fewer than 10 variants with DMS measurements for all genes in our analysis at an allele frequency cut‐off of 1%. Even with an allele frequency cut‐off of 0.01%, only *TP53*, *CBS*, *MTHFR* and *MSH2* would retain sufficient variants. Since the primary purpose of most VEPs is to assign labels to rare variants that are frequently identified through sequencing, it is potentially more informative to retain these variants in the putatively benign dataset, as has recently been discussed (Wu *et al*, 2021). Furthermore, as common and rare benign variants may have distinct features (Ioannidis *et al*, 2016), benchmarking against only common variants is likely to be less reflective of actual clinical utility.

We used these datasets to calculate the area under the receiver operating characteristic curve (AUROC) statistic, which is a common technique for summarising classifier performance. One advantage of using AUROC for this study is that our pathogenic and putatively benign variant sets are essentially independent of each other: the number of pathogenic variants for each gene will be influenced by the frequency of disease, and how closely it has been studied, while the number of putatively benign variants is determined by the individuals in gnomAD. The nature of AUROC means that it should be independent of the size of either variant dataset; for example, if we increased the size of our putatively benign dataset by considering a larger population cohort, or added more pathogenic variants to a particular gene due to more focussed sequencing studies, the AUROC should not change by much, unless the initial dataset was too small to adequately represent one of the classes.

We calculated AUROC for every protein with available DMS data and at least 10 pathogenic and 10 putatively benign missense variants. We also supplemented the *SNCA* dataset with additional variants from the literature (Kapasi *et al*, 2020; Fevga *et al*, 2021; Daida *et al*, 2022) and the *CALM1* dataset by adding variants from *CALM2* and *CALM3*, which have identical amino acid sequences.

**Figure 3:**
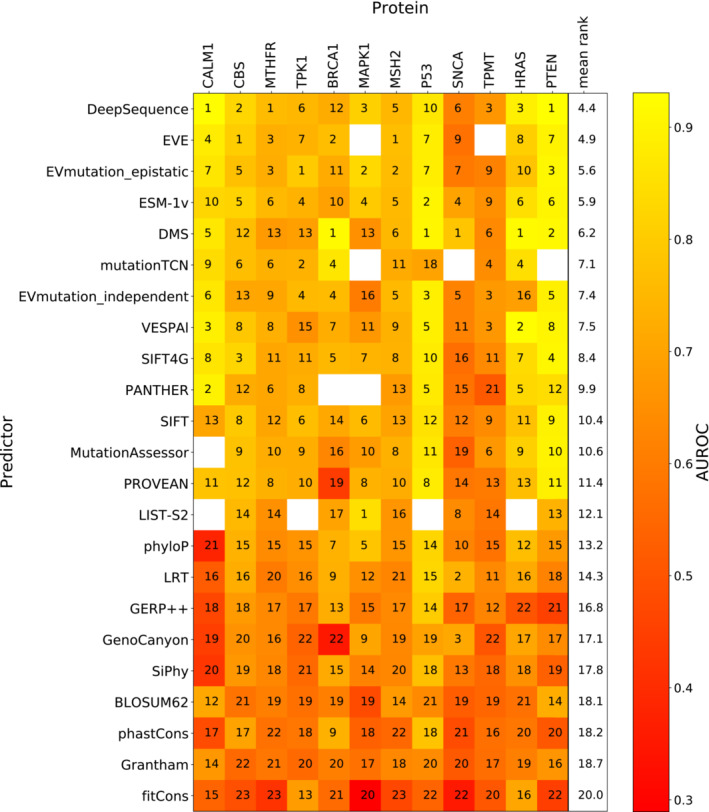
Ranking of DMS and unsupervised VEPs using clinical missense variants The rankings of DMS and unsupervised VEPs by AUROC. The colour scale represents the AUROC of each predictor for classifying pathogenic and putatively benign variants in every protein. The numbers indicate the relative ranking of all predictors for each protein while rank ties are assigned the same rank as the top‐ranking member of the group.

Similar to our ranking analysis, we compared the AUROC of every pair of unsupervised predictors or DMS score sets using only variants shared between them (providing at least 10 ClinVar and 10 gnomAD variants were shared). The method that produces the higher AUROC in each pairwise comparison gains one point. Figure 3 shows the rankings of each unsupervised predictor based on its mean rank across every protein. We selected the best‐ranking DMS score per protein to represent the overall DMS rankings. Similar to the correlation‐based analysis, we determined the relative significance of the ranking using a bootstrapping approach whereby pathogenic and benign variant were resampled with replacement 1,000 times and the ranking recalculated (Dataset EV1).

The DMS datasets showed highly heterogeneous performance, ranking first for *TP53*, *BRCA1*, *SNCA* and *HRAS* but performing somewhat poorly for *TPK1*, *MTHFR* and *MAPK1*. DMS ranked fifth by overall mean ranking across all proteins but was not a significantly lower performer than the top VEPs. The top three VEPs (DeepSequence, EVE and ESM‐1v) as well as DMS also do not differ significantly in ranking. We note that, although *TPK1* DMS outperformed all VEPs in our previous study, the inclusion of more pathogenic missense variants here (increasing from 8 to 15) has substantially affected its performance. The *TPK1* DMS data were also interesting for another reason: compared with the *CALM1* data from the same study, the *TPK1* scores were inverse predictors of clinical outcome (*i.e*. they produced an AUROC under 0.5). To maintain comparability, we inverted the scale of the *TPK1* scores.

**Figure EV2:**
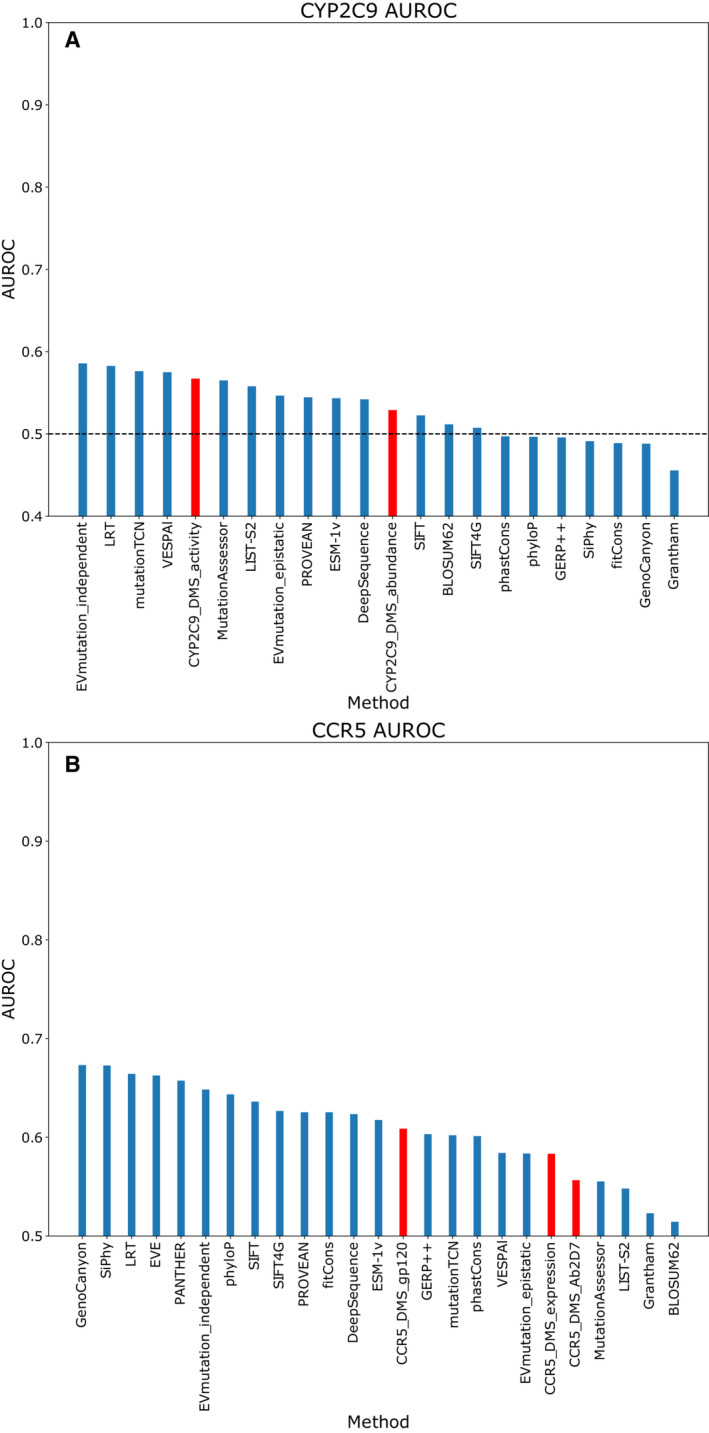
Performance of DMS and unsupervised VEPs for classifying variants in *CYP2C9* and *CCR5* The area under the balanced precision‐recall curve for DMS and unsupervised VEPs for classifying “pathogenic” ClinVar and HGMD variants in (A) *CYP2C9* and (B) *CCR5*.

While both *CYP2C9* and *CCR5* had enough apparently pathogenic variants to be included in this analysis, close inspection indicated that most of the HGMD variants were not truly pathogenic. *CYP2C9* is an enzyme involved in drug metabolism, and most variants in ClinVar and HGMD have an altered drug response phenotype. Using these variants as a “pathogenic” dataset for the purpose of calculating AUROC produces extremely poor results across all VEPs and DMS datasets (Fig EV2A). Another contributing factor is likely the presence of many drug response variants in gnomAD, which would not be filtered out. Using a specialised database such as PharmVar (Gaedigk *et al*, 2018) may be more appropriate for assessing the performance of VEPs and DMS datasets for variant interpretation in this protein. *CCR5* is a cell‐surface chemokine receptor expressed by T‐cells and macrophages. The protein is also important for HIV cell entry, and most ClinVar and HGMD records are variants that alter HIV binding affinity. While AUROC results support some modest ability of VEPs and DMS to identify these variants that affect HIV entry (Fig EV2B), they are not necessarily relevant to human genetic disease.

**Figure 4:**
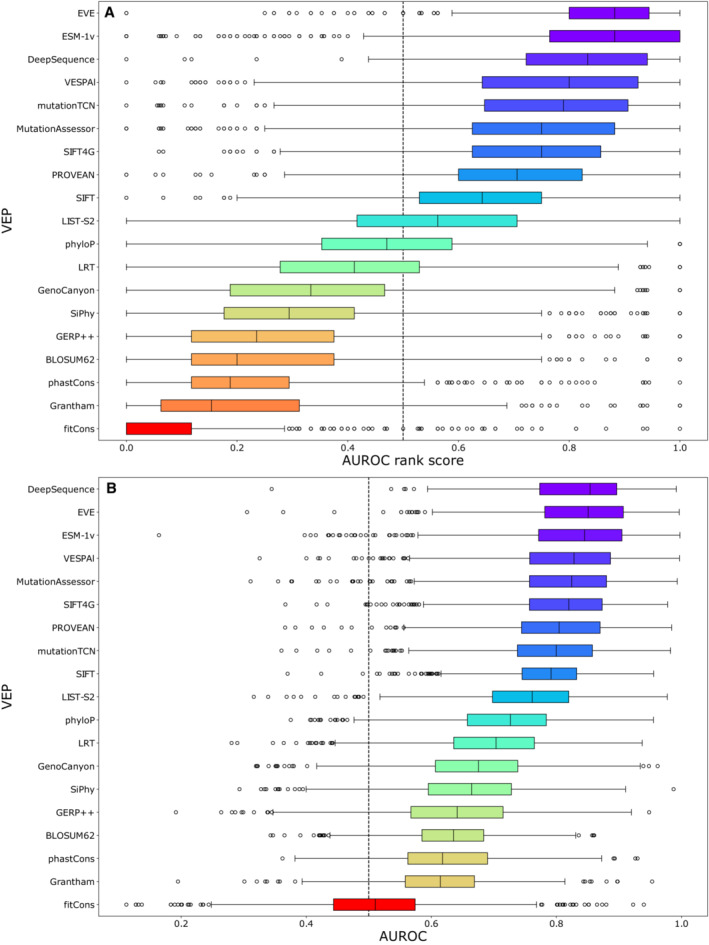
Performance of unsupervised VEPs against clinical missense variants The distribution of AUROC‐based rank scores for unsupervised VEPs on ClinVar and gnomAD variants from 985 proteins.Distribution of the raw AUROCs for each unsupervised VEP on ClinVar and gnomAD variants from 985 proteins. Outliers are plotted as individual points when they occur 1.5 times the interquartile range beyond the 1^st^ or 3^rd^ quartile. A black line indicates the median of each distribution. EVmutation is excluded from this analysis due to predictions being available for only a limited number of proteins.

**Figure EV3:**
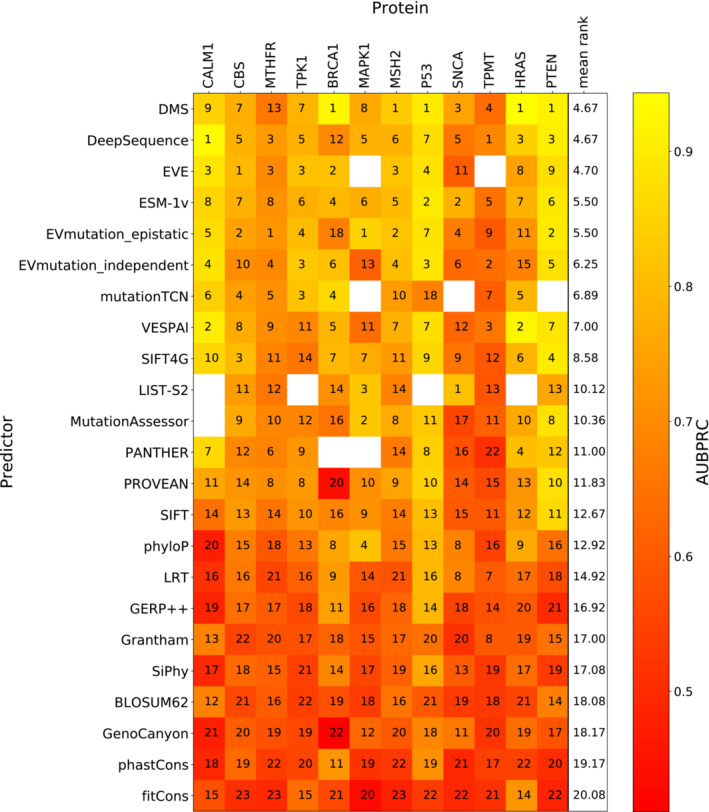
Ranking of DMS and unsupervised VEPs using clinical missense variants and AUBPRC The rankings of DMS and unsupervised VEPs by AUBPRC using shared variants. The colour scale of the heatmap represents the AUBPRC of each predictor for classifying pathogenic and putatively benign variants in every protein. The numbers indicate the relative ranking of all predictors for each protein while rank ties are assigned the same rank as the top‐ranking member of the group.

A common criticism of AUROC is that its insensitivity to class balance means it could be considered to overestimate performance in cases with few positive (pathogenic) samples compared with negative, which is the case for several proteins in our dataset. Precision‐recall curves can be useful in these situations as an alternate performance metric, where the focus is on correct prediction of the positive class; however, for the area under the precision‐recall curve to be comparable, the predictors need the same numbers of samples in both classes, which makes comparisons of different proteins difficult. As an alternative, we have also employed the area under the balanced precision‐recall curve (AUBPRC) statistic (Wu *et al*, 2021), which provides the advantages of precision‐recall while remaining comparable across datasets with differing class balances. When calculated using AUBPRC, DMS improves in overall ranking to joint first (with DeepSequence) and becomes the top predictor for *MSH2* and *PTEN*, but loses *SNCA* (Fig EV3), although it remains statistically indistinguishable from DeepSequence, EVE, EVmutation and ESM‐1v. The strong performance of DMS when assessed using the AUBPRC metric suggests that DMS may be generally useful for identifying clinically relevant variants, but the relatively poorer performance with AUROC shows that DMS may be weaker than some VEPs at correctly classifying benign variants.

### Benchmarking unsupervised VEPs on large clinical datasets

The issues of type 1 and 2 data circularity apply primarily to supervised VEPs; in contrast, unsupervised VEP predictions cannot be overtly influenced in the same way, as these methods are not trained using labelled data although biases may still emerge based on the composition of the underlying data (often a MSA). It is also possible that some unsupervised VEPs are tweaked based on performance against clinical observations that could reintroduce type 1 circularity into performance assessments but in general, we consider unsupervised VEPs immune to these forms of bias. As data circularity is far less likely in unsupervised VEPs, the use of traditional benchmarks with clinical data for these methods is likely to be a much better reflection of actual performance than for supervised VEPs. Therefore, to assess the performance of all unsupervised VEPs against clinical data on a large scale, we identified all human proteins with at least 10 pathogenic or likely pathogenic missense variants in ClinVar, and 10 other missense variants in gnomAD, leaving us 985 proteins. Where possible, we obtained predictions from 18 unsupervised VEPs for all variants in these proteins. To compensate for the fact that some VEPs were unable to make predictions for all missense variants in a protein, we again used a pairwise ranking approach, whereby every pair of unsupervised VEPs were compared by AUROC and calculated the significance of the ranks by bootstrapping (Dataset EV1). Figure 4A shows the distributions of rank scores for unsupervised predictors across all proteins.

**Figure EV4:**
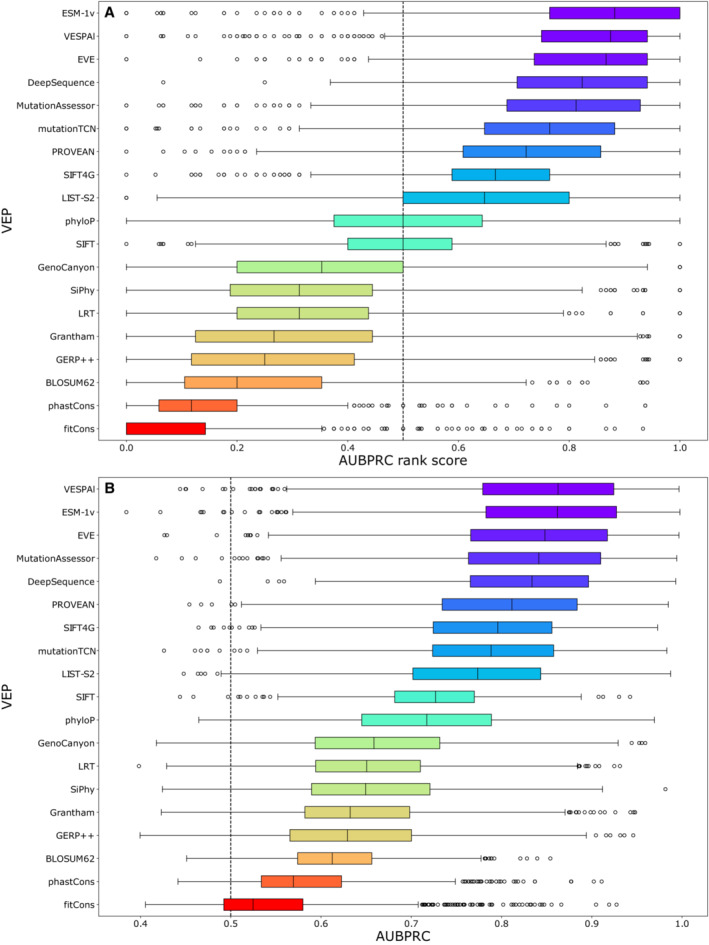
Performance of unsupervised VEPs against clinical missense variants by AUBPRC The distribution of AUBPRC‐based rank scores for unsupervised VEPs on ClinVar and gnomAD variants from 985 proteins.Distribution of the raw AUBPRC for each unsupervised VEP on ClinVar and gnomAD variants from 985 proteins. Outliers are plotted as individual points when they occur 1.5 times the interquartile range beyond the 1^st^ or 3^rd^ quartile. A black line indicates the median of each distribution. EVmutation is excluded from this analysis due to predictions being available for only a limited number of proteins.

The top‐performing unsupervised VEPs by median rank score were EVE, ESM‐1v and DeepSequence, which all produced median AUROC values in excess of 0.84 across all proteins (Fig 4B). EVE significantly outranked all other methods except ESM‐1v (*P* = 0.123), while ESM‐1v and DeepSequence were also not significantly distinct (*P* = 0.070). Overall, the results obtained by ranking unsupervised VEPs against clinical data were similar to their relative rankings against the DMS data with the largest difference being VESPAl, which ranked fourth using the clinical data compared with ninth out of the unsupervised VEPs against the DMS benchmark. Nucleotide conservation metrics and substitution matrices are relatively poor predictors of clinical effects, while the top five VEPs are all based on advanced unsupervised machine learning methodology. It has been noted that nucleotide‐based alignments (such as those that form the basis of GERP++, SiPhy and PhyloP) may be noisier than protein alignments (Wernersson & Pedersen, 2003) and that protein‐based alignments allow for more distantly related sequences to be included in the alignment (Pearson, 2013). Given recent advances in the alignment of biological sequence data, it is unclear to what extent these limitations of nucleotide alignments still apply, but this remains a possible contributor to the marked underperformance of nucleotide‐based predictors. Performing the same analysis using AUBPRC instead of AUROC (Fig EV4A and B) gives very similar results, although ESM‐1v ranks first but is not significantly different from VESPAl (*P* = 0.205) or EVE (*P* = 0.082).

**Figure 5:**
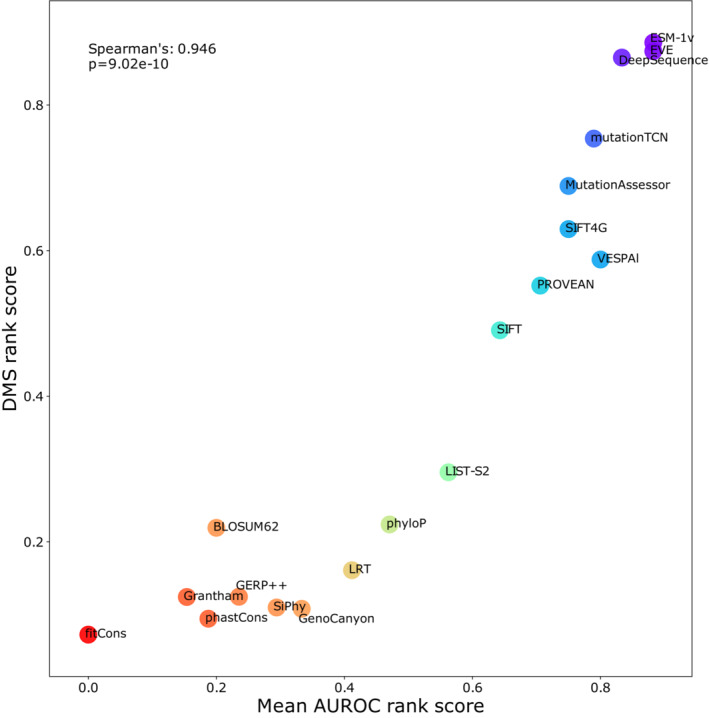
Relationship between correlation‐based rank score and AUROC‐based rank score for unsupervised VEPs The rank score of unsupervised VEPs from Fig 2 plotted against AUROC‐based rank score from Fig 4A. The identity of each unsupervised VEP is indicated on the chart.

**Figure EV5:**
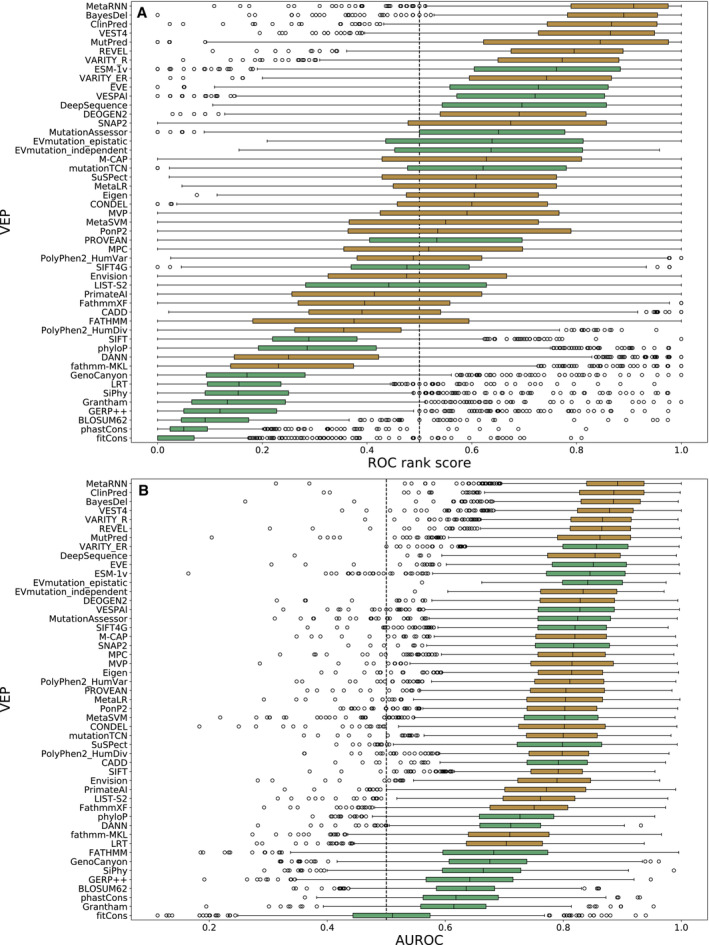
Performance of all VEPs against clinical missense variants The distribution of AUBPRC‐based rank scores for all VEPs on ClinVar and gnomAD variants from 985 proteins.Distribution of the raw AUBPRCs for all VEPs on ClinVar and gnomAD variants from 985 proteins. Bars are colour‐coded green for unsupervised VEPs and brown for supervised VEPs. Outliers are plotted as individual points when they occur 1.5 times the interquartile range beyond the 1^st^ or 3^rd^ quartile. A black line indicates the median of each distribution. EVmutation is excluded from this analysis due to predictions being available for only a limited number of proteins. The scale of Fathmm is inverted in this figure due to improved predictive performance.

While only unsupervised VEPs can be benchmarked in a fair manner using this approach, we can include the supervised VEPs in this analysis out of interest (Fig EV5A). In this comparison, the seven top‐ranked VEPs are supervised and the top three are all metapredictors that integrate multiple other VEPs as predictive features, which are thus capable of importing further bias from their component predictors. Importantly, the extent to which data circularity influences the performance of each supervised VEP cannot be reliably ascertained, so we do not believe that the relative rankings or AUROCs (Fig EV5B) of supervised VEPs observed here are particularly meaningful. Despite this advantage, recent unsupervised methods remain competitive with many supervised predictors on large clinical datasets.

**Figure EV6:**
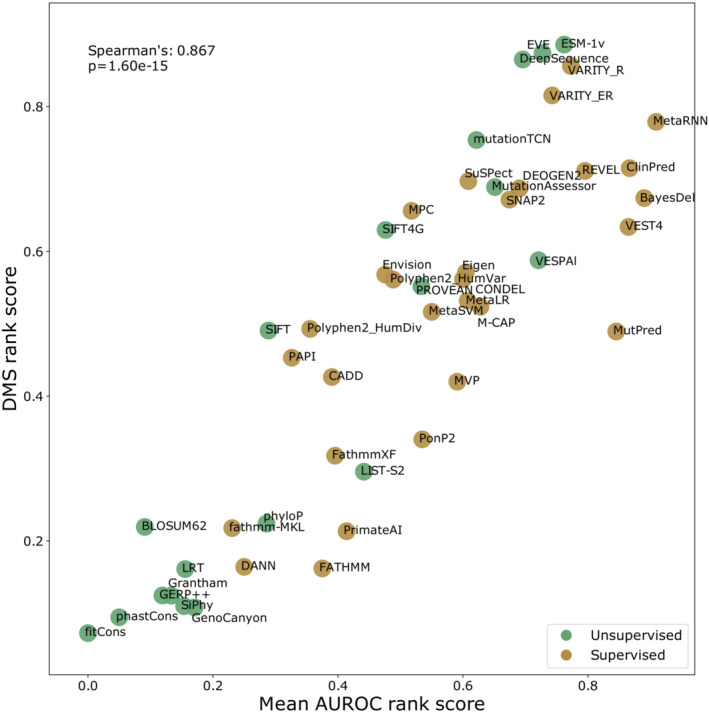
Relationship between correlation‐based rank score and AUROC‐based rank score for all VEPs The rank score of VEPs from Fig 2 plotted against the AUROC‐based rank score from Fig EV5A. The identity of each VEP is indicated on the chart, and points are coloured green for unsupervised and brown for supervised VEPs. The scale of Fathmm is inverted in this figure for AUROC calculation due to improved predictive performance.

Comparison of the rank score obtained by benchmarking of unsupervised VEPs with DMS data and the rank score obtained by using AUROC on large clinical datasets demonstrates remarkable agreement (Spearman's correlation: 0.946, *P* = 9.02 × 10^−10^), strongly supporting the utility of both independent benchmarking strategies (Fig 5). Adding supervised VEPs to this analysis identifies those predictors that overperformed at the clinical benchmark relative to the DMS benchmark and are thus more likely to have been influenced by data circularity (Fig EV6). MetaRNN, ClinPred, BayesDel, VEST4 and MutPred in particular show much better performance on the clinical data than the DMS data.

## Discussion

Our updated analysis produced some interesting results in terms of predictor ranking; DeepSequence remained a highly ranked method, but was joined by ESM‐1v, EVE and VARITY. The strong performance of many new predictors indicates that VEP methodologies are continuing to improve. In our previous study, supervised VEPs were previously superior to most unsupervised methods, with the exception of DeepSequence. Our present results indicate that most of the top‐10 VEPs are now unsupervised, demonstrating that multiple unsupervised methodologies are viable for VEP development and that researchers are taking the potential for bias seriously and making efforts to avoid introducing it into new VEPs. No particular machine learning technique is dominant among the top‐ranking VEPs, indicating that multiple approaches to variant effect prediction remain powerful with their unique advantages and disadvantages.

The excellent performance of ESM‐1v is particularly interesting, not due to its nature as an unsupervised VEP, but because it had no access to a protein‐specific multiple sequence alignment like EVE, DeepSequence and EVmutation. While type 1 and 2 data circularity poses no issue for these predictors, sampling bias from the database used to construct MSAs still has the potential to influence predictions in some proteins. ESM‐1v has demonstrated that even this source of bias can be mitigated, although not entirely eliminated, as language models are still trained using a sequence database, albeit a very large and varied one. We used ESM‐1v in a zero‐shot setting, where no MSA generation took place, but it is also possible to use the model in a “few‐shot” setting, where a protein‐specific MSA is provided to assist with protein‐specific predictions. The authors of the method found that using the model in a few‐shot context improved predictions slightly (preprint: Meier *et al*, 2021) but we were unable to successfully run this model on our system.

For the supervised methods Envision and VARITY, this analysis does not constitute a truly independent benchmark, as some of the DMS datasets from our benchmark were also a part of their training data. VARITY may be somewhat optimised for predicting the results of DMS experiments in general, but its strong performance on datasets that were not used in its training suggests that this is not a major issue. It seems likely that more newly developed VEPs will incorporate DMS data in the future. While it makes little sense to exclude DMS datasets as a potential source of training data, it does mean that future benchmarking using this data may carry the same caveats as benchmarking supervised predictors using variant databases. Similar scenarios will likely arise for any new source of benchmarking data, as it will eventually be used as a training dataset for new VEPs. We must continue to be vigilant regarding the data used to train VEPs, and where possible ensure that fully independently derived data are used for benchmarking.

Our analysis demonstrates that unsupervised methods excel when benchmarked against independent DMS data. In contrast, when assessed against human pathogenic and putatively benign missense variants, certain supervised VEPs outperform the top unsupervised methods, but this is almost certainly influenced by data circularity. Nevertheless, it is essential to consider that the superior performance of supervised methods on clinical variants may also stem from their specific design and optimisation for this particular purpose, rather than the more general task of predicting functional effects across all possible variants, which is assessed by the DMS benchmark. Future research and clever design of unbiased clinical benchmarks will be needed to disentangle this difficult issue.

Several DMS datasets demonstrated consistently low correlations with VEP predictions. While the systematic nature of the poor correlations appeared to indicate that in these cases the DMS study design was not sufficiently related to the human disease mechanisms to accurately recapitulate disease‐related fitness effects that may not always be the case. Our group has recently demonstrated that VEPs consistently underperform on non‐loss‐of‐function mutations, in particular dominant‐negative and gain‐of‐function (Gerasimavicius *et al*, 2022). On a structural level, both dominant‐negative and gain‐of‐function mutants tend to be less structurally disruptive than loss‐of‐function. This may be the case for *SNCA* where gain‐of‐function can lead to fibril formation (Bertoncini *et al*, 2005) and in *CALM1* where the dominant‐negative effect has been observed (Rocchetti *et al*, 2017). The tendency of *CALM1* mutants to be dominant‐negative raises a further issue, which is that in the yeast growth‐rate‐based DMS assay assessing the performance of *CALM1* mutants, pathogenic dominant‐negative mutations would likely score as less damaging than null mutants. This could result in neither DMS nor VEPs from picking up on pathogenic mutations, despite agreeing.

**Figure EV7:**
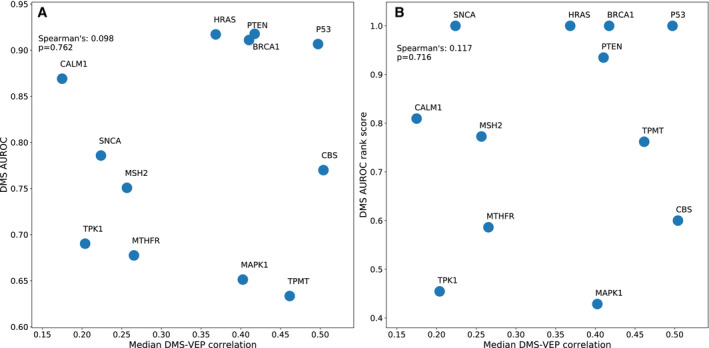
Relationship between median VEP‐DMS correlation and AUROC The median correlation between each DMS dataset and all VEPs plotted against the AUROC of each DMS dataset.The median correlation between each DMS dataset and all VEPs plotted against the AUROC‐based rank score of each DMS dataset.

Our AUROC analysis included six further proteins over our previous study and made use of numerous additional variants deposited in ClinVar since 2018 and HGMD. While DMS data did not perform as the top predictor for the majority of proteins, it was still often among the top methods. Notably, DMS ranked first for four proteins, which was more than any individual VEP. However, DMS also performed quite poorly for some proteins, demonstrating that DMS datasets are heterogeneous in their performance in disease variant classification. We previously claimed that DMS experiments based on growth rate tended to be more representative of human disease mutations compared with those based on protein expression levels or other assays. With an expanded set of DMS data and additional variants, this conclusion no longer seems valid as some DMS assays based on expression levels and quantifying protein–protein interactions predicted disease as well as those based on yeast complementation or general growth rate. It is crucial that we learn what factors make a DMS dataset reliable for this purpose, whether they be related to the target protein specifically, the choice of experimental assays, or other technical experimental issues. Is there some way we can predict *a priori* whether a DMS dataset will be predictive of variant pathogenicity? Interestingly, there is little correspondence between the median VEP correlation with DMS datasets and the performance of DMS datasets for variant classification in terms of AUROC (Fig EV7A) or pairwise ranking (Fig EV7B). However, it is notable that the most clinically predictive datasets were all for cancer‐related genes (*P53*, *BRCA1*, *PTEN* and *MSH2* as tumour suppressors and *HRAS* as a proto‐oncogene), all of which except *MSH2* also have relatively high correlations with VEP predictions. It may be that the observed growth rate changes in these DMS studies are more reflective of the actual functional changes seen in human disease than for other classes of genes.

Finding suitable benchmarks to compare VEPs is an ongoing challenge for the field of variant effect prediction, particularly when many of those VEPs are supervised. In addition to DMS datasets, other suitable sources of data that may yield equally bias‐free results exist. Prediction performance on case–control disease studies would also not be reliant on existing clinical labels, but would greatly reduce the diversity of variants tested (McInnes *et al*, 2019; Wu *et al*, 2021). This approach can also be scaled up and applied to multiple relevant gene‐trait combinations (preprint: Kuang *et al*, 2022).

Our results continue to indicate that benchmarking using independent variant effect datasets is a powerful strategy for reducing data circularity when assessing VEP performance. The potential of DMS for direct variant effect prediction remains an exciting option, although care should be taken to ensure that the assay used is indicative of phenotypic outcome. With just 2 years' worth of additional data, we more than doubled the amount of DMS datasets in this analysis, and it is likely that with projects like the Atlas of Variant Effects (www.varianteffect.org), the availability of such datasets, and their utility for protein variant interpretation, will explode.

## Materials and Methods

### DMS identification and criteria

We retained 13 DMS datasets in human proteins from our previous analysis and identified a further 19 studies with publically available datasets from MAVEDB (Esposito *et al*, 2019; https://www.mavedb.org/) and literature searches. We applied a threshold of 5% minimum coverage of all amino acid variants within the target protein to prevent any particularly low‐coverage studies from skewing our results. This prevented a *SCN5A* dataset being included (Glazer *et al*, 2020). We also excluded datasets for *NCS1* and *TECR* obtained from MAVEDB as no methodology was published with them at the time.

### VEP score retrieval

Most VEP predictions were retrieved from the dbNSFP database version 4.2 (academic; Liu *et al*, 2020). Scores were retrieved for the transcript that matches the canonical Uniprot sequence for each protein. As dbNSFP is a nucleotide‐resolution database, there are instances where multiple nucleotide variants map to the same amino acid substitution. In these cases, the mean of the VEP scores mapping to the same substitution was used.

SIFT was run locally using the UniRef90 database (Suzek *et al*, 2015) to generate multiple sequence alignments.

EVmutation scores were obtained from the EVcouplings pipeline (mutation stage). We used the Uniref100 database to generate alignments and default settings as found in: https://github.com/debbiemarkslab/EVcouplings/blob/develop/config/sample_config_monomer.txt except changing the minimum_column_coverage setting to 20 to reduce large alignment gaps.

DeepSequence was run locally using alignments generated by the EVcouplings pipeline with default settings. EVE results were partially retrieved online from: https://evemodel.org/ and others were run locally using default settings on a GPU and the same alignments as DeepSequence.

ESM‐1v results were obtained by adapting the example at: https://github.com/facebookresearch/esm/blob/main/examples/variant-prediction/predict.py and running locally on a GPU. The final score is the mean of esm1v_t33_560_UR90S_1, esm1v_t33_560_UR90S_2, esm1v_t33_560_UR90S_3, esm1v_t33_560_UR90S_4 and esm1v_t33_560_UR90S_5 outputs.

Sources for all VEPs can be found in Table EV4.

### Correlation analysis

For each protein we had DMS data for, we selected a single DMS dataset to be representative of it in our analysis. We selected the dataset with the highest median Spearman's correlation to all VEP predictions for that protein to help prevent outliers from influencing the choice of set.

Spearman's correlation was calculated using the scipy.stats.spearmanr() function of the python scipy package.

### Variant identification

For calculation of AUROC and AUBPRC values, we used pathogenic and likely pathogenic variants for the ClinVar database of clinically relevant variants (September 2022 update; Landrum *et al*, 2014; https://www.ncbi.nlm.nih.gov/clinvar/) and also from HGMD (public version; Stenson *et al*, 2003; https://www.hgmd.cf.ac.uk/ac/index.php) for those proteins we had DMS data for. ClinVar entries were filtered to only include variants with a one‐star annotation level or higher (assertion criteria provided). Additional pathogenic variants from *SNCA* were found through a literature search (Kapasi *et al*, 2020; Fevga *et al*, 2021; Daida *et al*, 2022). Variants in *CALM2* and *CALM3* were used to supplement *CALM1* variants as all three proteins share the same primary structure, although they differ at the genomic level.

We used the gnomAD database version 2.1.1 (Karczewski *et al*, 2020; https://gnomad.broadinstitute.org/) as a source of putatively benign variants. While these variants certainly contain some recessive and low‐penetrance pathogenic variants, gnomAD filters out individuals with severe paediatric disease and their first‐degree relatives. This means that gnomAD should be depleted in pathogenic variants relative to the population and serves as a useful estimate of benign variation. We performed no filtering based on allele frequency but only used variants that passed the gnomAD internal quality filters (inbreeding coefficient < −0.3, at least one sample with depth ≥ 10, genotype quality ≥ 20 and minor allele balance > 0.2).

### AUROC calculation

ROC AUC values were calculated using the sklearn.metrics.roc_auc_score() function of the sklearn python package. Pathogenic variants were labelled as true positives, and putatively benign variants were labelled as true negatives. Where VEPs or DMS had an inverted scale (lower scores being more pathogenic), the scores were converted to a comparable scale using:

$$\text{Modified score}=¦\text{score}\;-\;\max\left(\text{score}\right)¦$$

### AUBPRC calculation

Precision‐recall AUC was calculated using the sklearn.metrics.average_precision_score() function of the sklearn python package. Average precision uses a weighted mean of precision scores at each threshold of the precision‐recall curve to summarise the curve (Turpin & Scholer, 2006). Pathogenic samples were labelled as true positives, while putatively benign samples were labelled true negatives. Predictors with inverted scores were modified as with the AUROC calculation.

Average precision scores were then converted to balanced average precision using the formula presented by (Wu *et al*, 2021)

$$\text{AUBPRC}=\frac{\text{AUPRC}*\left(1-\text{prior}\right)}{\text{AUPRC}*\left(1-\text{prior}\right)+\left(1-\text{AUPRC}\right)*\text{prior}}$$

Where the prior is the proportion of positive (in this case pathogenic) samples and AUPRC is the area under the precision‐recall curve (for which we used average precision).

AUBPRC can be interpreted as the precision‐recall AUC if the classes were balanced, which removes the main disadvantage of precision‐recall of being incomparable if the balance of sample labels changes.

### Rank score calculation

The rank scores presented in Figs 2 and 3 were calculated using a series of pairwise comparisons, ensuring that only data shared between the predictors being compared were used. For the rank score based on DMS‐VEP correlation in Fig 2, for each protein the Spearman's correlation between each pair of predictors and the DMS data was calculated using only amino acid substitutions shared between the three methods. The “winning” VEP in every comparison gains one point. The total points scored by each VEP are then divided by the number of tests it participated in, generating a per‐protein rank score. Finally, the mean of this score is taken for each VEP across all proteins to generate the final rank score.

The AUROC‐based rank score in Fig 3 was calculated using similar methodology. AUROC was compared for every pair of VEPs/DMS datasets for each protein using only variants that were shared between the methods. The “winning” predictor from each comparison was awarded one point. The total points scored by each predictor were then divided by the number of tests it participated in generating a per‐protein rank score. The final rank score is the mean of the per‐protein score across all proteins. Only the top‐scoring DMS dataset per‐protein was taken to represent DMS in the final results. The same strategy was used to calculate the balanced AUBPRC‐based rank scores in Fig EV3.

### Bootstrapping

Significance of the rankings was calculated using a bootstrapping methodology. The data shared between VEPs were randomly resampled with replacement 1,000 times and used to calculate a new ranking. The total number of times that each VEP outranked every other VEP in these 1,000 iterations indicates the significance of the rank values, with a total of 950 being equivalent to a *P*‐value of 0.05 and indicating statistical significance. For ROC‐based analyses, the pathogenic and putatively benign datasets were sampled independently to ensure that no situations arose where one class was fully removed from the analysis.

## Author contributions

**Benjamin J Livesey:** Data curation; formal analysis; investigation; writing – original draft. **Joseph A Marsh:** Conceptualization; supervision; funding acquisition; writing – review and editing.

## Disclosure and competing interests statement

The authors declare that they have no conflict of interest.

## Supporting information



Expanded View Figures PDF

Table EV1

Table EV2

Table EV3

Table EV4

Table EV5

Table EV6

Dataset EV1

Dataset EV2

Dataset EV3

Dataset EV4

PDF+

## Data Availability

A compiled dataset of all DMS scores and VEP predictions used to perform this analysis is available at: https://doi.org/10.6084/m9.figshare.21581823.v1.
