# Supplementary material for: Updated benchmarking of variant effect predictors using deep mutational scanning
Source: Mol Syst Biol. 2023 Jun 13;19(8):e11474. doi: 10.15252/msb.202211474 (PMC10407742; doi:10.15252/msb.202211474)
Supplement: Supplementary file 1 — Expanded View Figures PDF [file MSB-19-e11474-s012.pdf]

Expanded View Figures

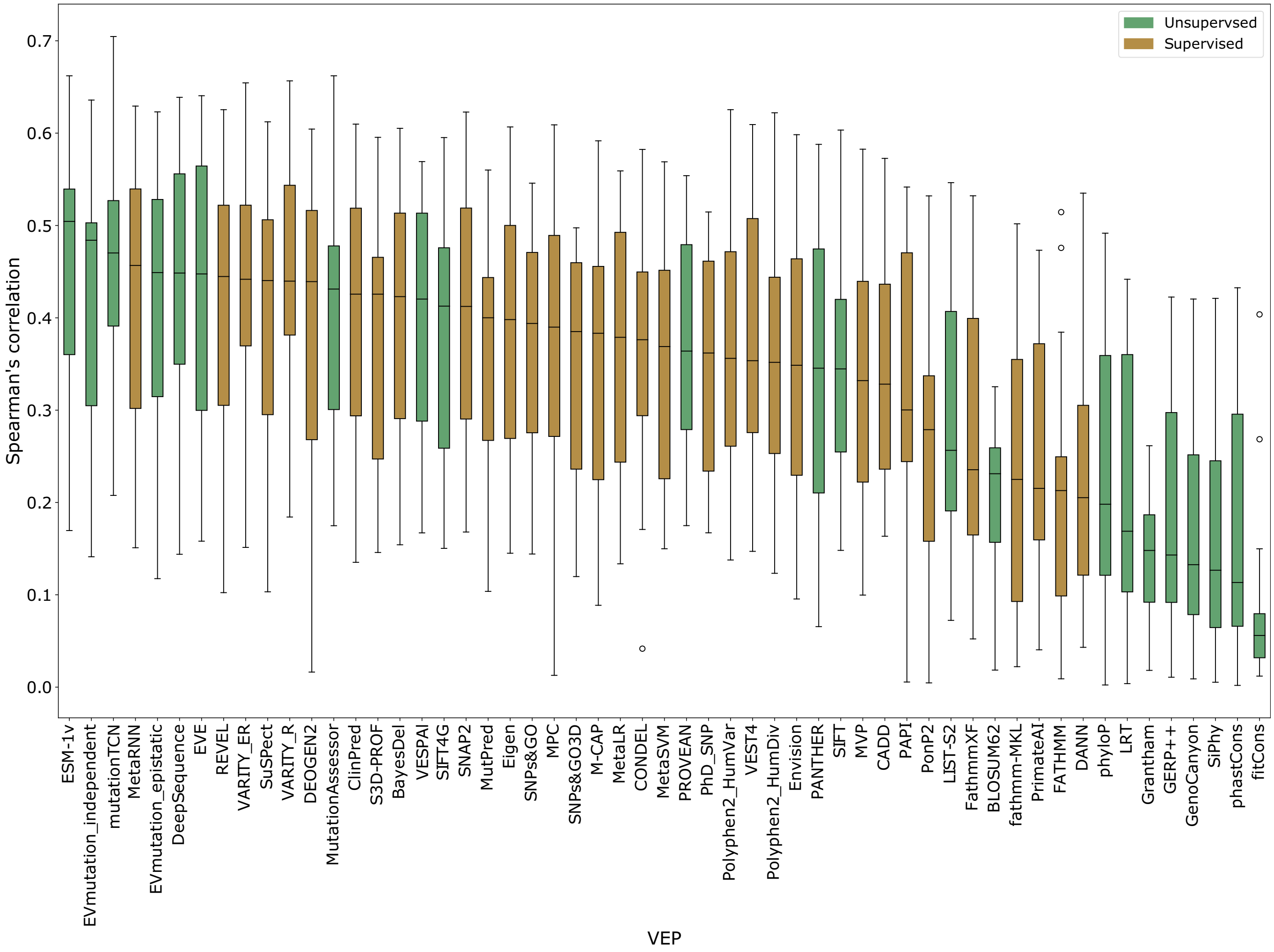

Figure EV1. Distribution of Spearman's correlations between VEPs and DMS datasets.

The distribution of Spearman's correlations between all VEPs and each selected DMS dataset ranked by the median correlation (black bar). The boxplot whiskers indicate the range of the data while flier points are represented by empty circles.

 © 2023 The Authors

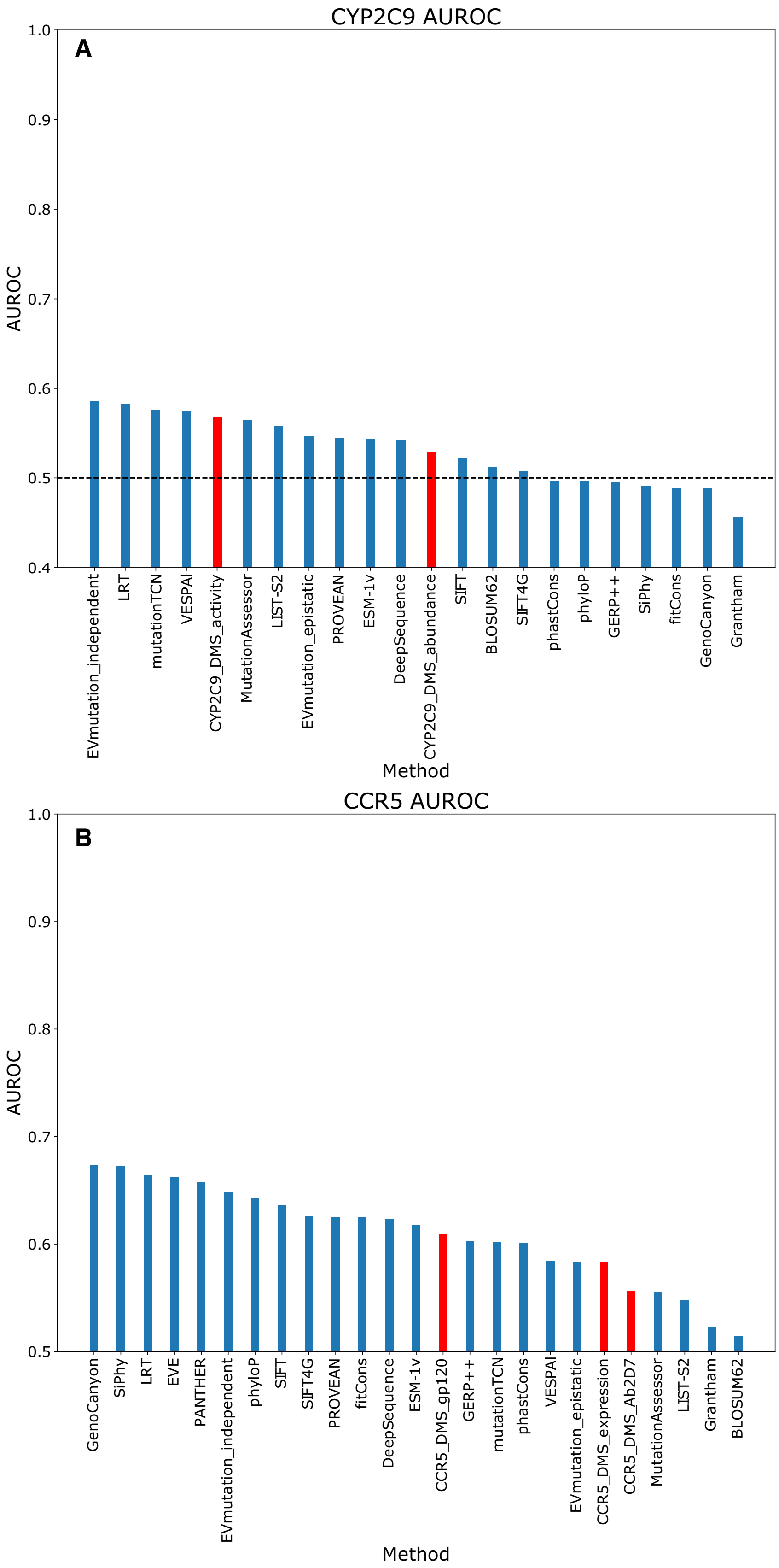

Figure EV2. Performance of DMS and unsupervised VEPs for classifying variants in *CYP2C9* and *CCR5*.

A, B The area under the balanced precision-recall curve for DMS and unsupervised VEPs for classifying “pathogenic” ClinVar and HGMD variants in (A) *CYP2C9* and (B) *CCR5*.

© 2023 The Authors

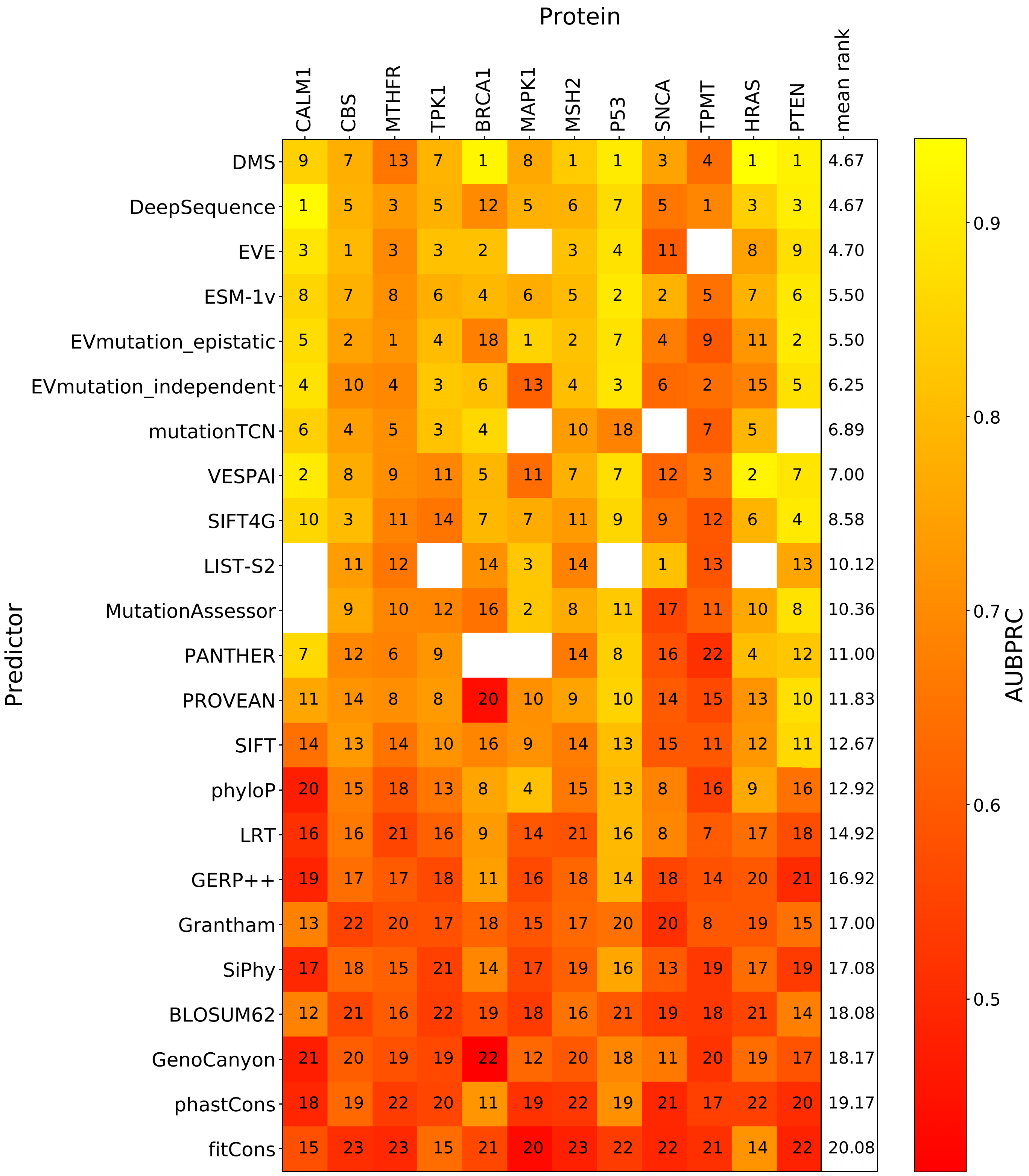

Figure EV3. Ranking of DMS and unsupervised VEPs using clinical missense variants and AUBPRC.

The rankings of DMS and unsupervised VEPs by AUBPRC using shared variants. The colour scale of the heatmap represents the AUBPRC of each predictor for classifying pathogenic and putatively benign variants in every protein. The numbers indicate the relative ranking of all predictors for each protein while rank ties are assigned the same rank as the top-ranking member of the group.

 © 2023 The Authors

Figure EV4. Performance of unsupervised VEPs against clinical missense variants by AUBPRC.

A The distribution of AUBPRC-based rank scores for unsupervised VEPs on ClinVar and gnomAD variants from 985 proteins.

B Distribution of the raw AUBPRC for each unsupervised VEP on ClinVar and gnomAD variants from 985 proteins. Outliers are plotted as individual points when they occur 1.5 times the interquartile range beyond the 1st or 3rd quartile. A black line indicates the median of each distribution. EVmutation is excluded from this analysis due to predictions being available for only a limited number of proteins.

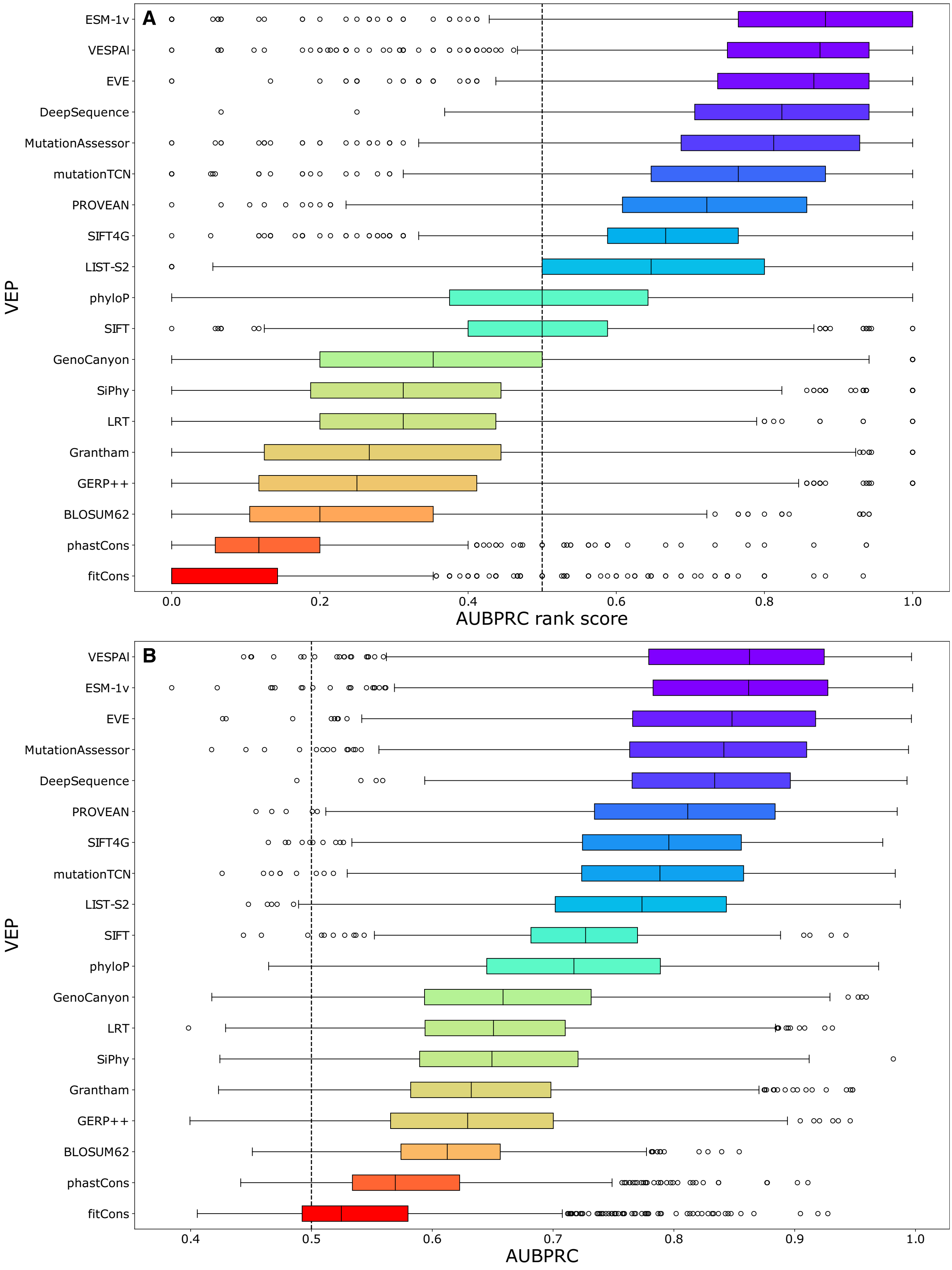

Figure EV4.

 © 2023 The Authors

Figure EV5. Performance of all VEPs against clinical missense variants.

A The distribution of AUBPRC-based rank scores for all VEPs on ClinVar and gnomAD variants from 985 proteins.

B Distribution of the raw AUBPRCs for all VEPs on ClinVar and gnomAD variants from 985 proteins. Bars are colour-coded green for unsupervised VEPs and brown for supervised VEPs. Outliers are plotted as individual points when they occur 1.5 times the interquartile range beyond the 1st or 3rd quartile. A black line indicates the median of each distribution. EVmutation is excluded from this analysis due to predictions being available for only a limited number of proteins. The scale of Fathmm is inverted in this figure due to improved predictive performance.

© 2023 The Authors

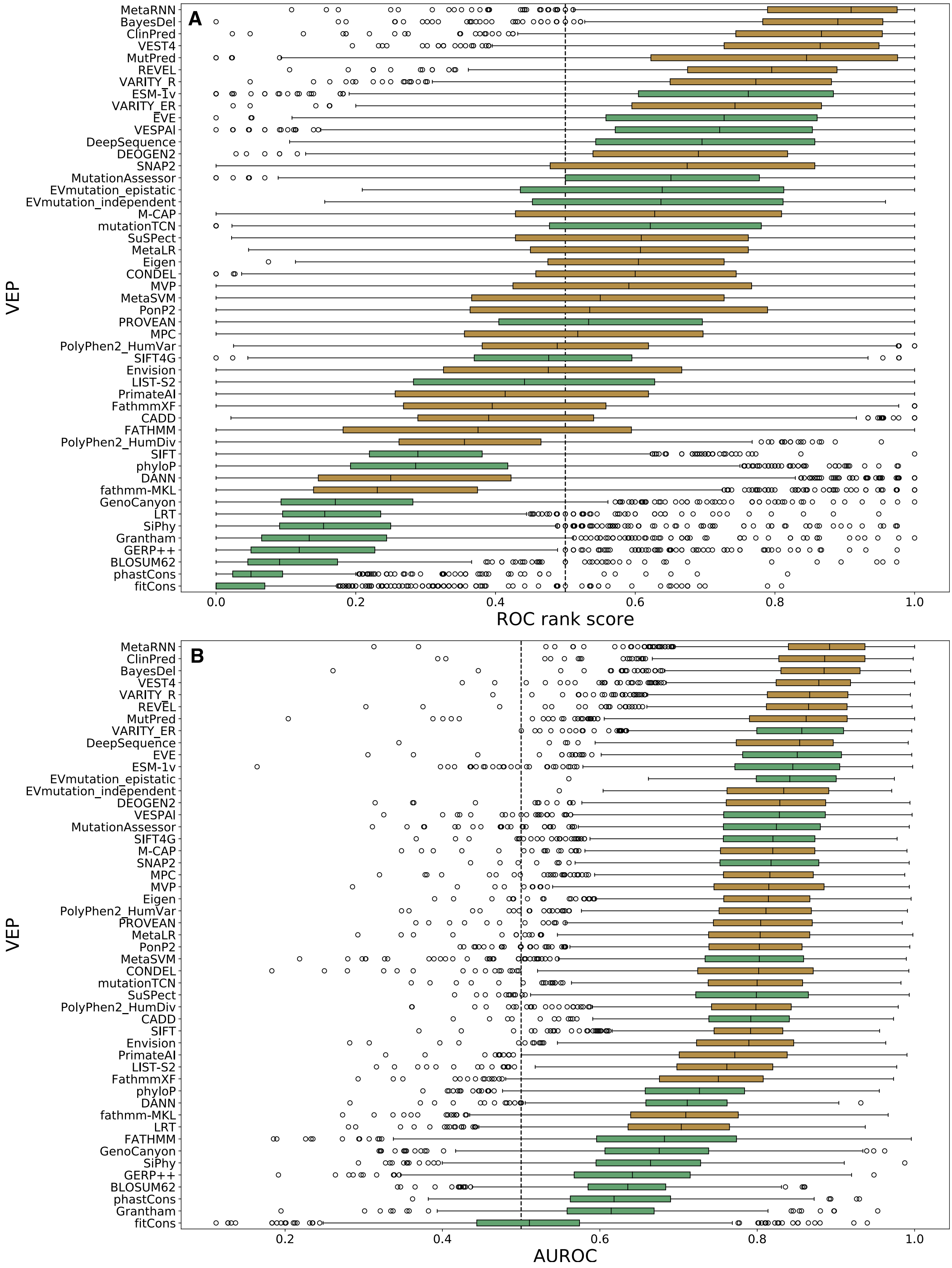

Figure EV5.

 © 2023 The Authors

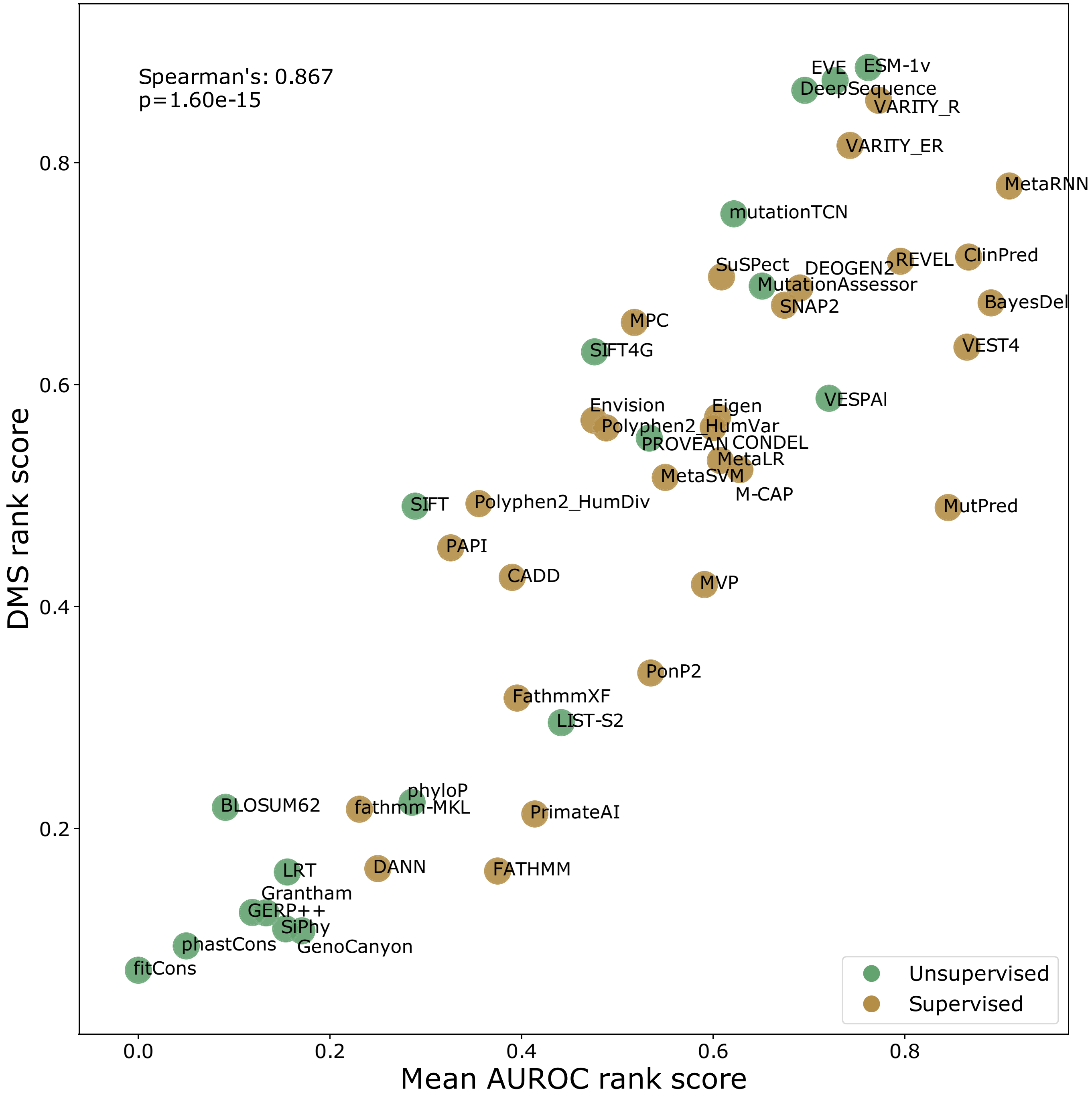

Figure EV6. Relationship between correlation-based rank score and AUROC-based rank score for all VEPs.

The rank score of VEPs from Fig 2 plotted against the AUROC-based rank score from Fig EV5A. The identity of each VEP is indicated on the chart, and points are coloured green for unsupervised and brown for supervised VEPs. The scale of Fathmm is inverted in this figure for AUROC calculation due to improved predictive performance.

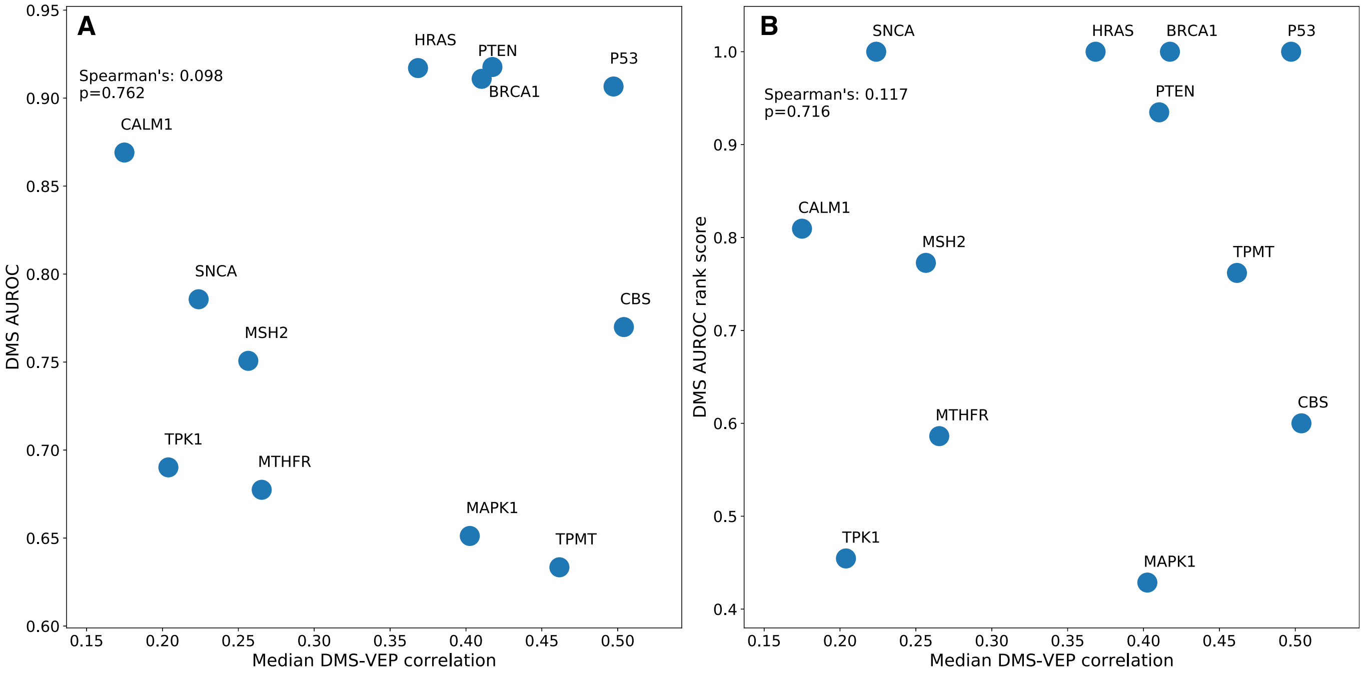

Figure EV7. Relationship between median VEP-DMS correlation and AUROC.

A The median correlation between each DMS dataset and all VEPs plotted against the AUROC of each DMS dataset.

B The median correlation between each DMS dataset and all VEPs plotted against the AUROC-based rank score of each DMS dataset.

 © 2023 The Authors
