# Supplementary material for: Updated benchmarking of variant effect predictors using deep mutational scanning
Source: Mol Syst Biol. 2023 Jun 13;19(8):e11474. doi: 10.15252/msb.202211474 (PMC10407742; doi:10.15252/msb.202211474)
Supplement: Supplementary file 2 — Table EV1 [file MSB-19-e11474-s005.docx]

| **Protein (Uniprot)** | **Functional assay** | **Selected score (internal name)** | **Percentage coverage of all amino acid variants (%)** | **Reference** |
| --- | --- | --- | --- | --- |
| ***CALM1*** (P0DP23) | Competitive growth assay in yeast | DMS_flipped | 64.04 | (Weile *et al*, 2017) |
| ***TPK1*** (Q9H3S4) |  | DMS_flipped | 68.90 |  |
| ***SUMO1*** (P63165) |  | DMS_flipped | 88.59 |  |
| ***UBE2I*** (P63279) |  | DMS_flipped | 85.38 |  |
| ***CBS*** (P35520) | Competitive growth assay in yeast | DMS_refined_lowB6 | 64.41 | (Sun *et al*, 2020) |
| ***GDI1*** (P31150) | Competitive growth assay in yeast | DMS | 51.40 | (Silverstein *et al*, 2021) |
| ***HMGCR*** (P04035) | Competitive growth assay in yeast | DMS_no_statin | 99.89 | (Jiang, 2019) |
| ***LDLRAP1*** (Q5SW96) | Yeast two-hybrid | DMS_OBFC1 | 99.03 |  |
| ***MTHFR*** (P42898) | Competitive growth assay in yeast | DMS_25_A222V | 99.85 | (Weile *et al*, 2021) |
| ***BRCA1***(a) (P38398) | Yeast two-hybrid and phage display. | N/A | N/A | (Starita *et al*, 2015) |
| ***BRCA1***(b) (P38398) | Growth rate of HAP1 cells | DMS_b | 5.19 | (Findlay *et al*, 2018) |
| ***MAPK1*** (P28482) | Growth rate of A375 cells | DMS_DOX | 99.56 | (Brenan *et al*, 2016) |
| ***MSH2*** (P43246) | Rescue of MMR-deficient HAP1 cells | DMS | 94.38 | (Jia *et al*, 2021) |
| ***NUDT15*** (Q9NV35) | Drug resistance assay. | DMS_sensitivity | 94.16 | (Suiter *et al*, 2020) |
| ***P53***(a) (P04637) | Growth assay in the presence of P53 agonists | N/A | N/A | (Giacomelli *et al*, 2018) |
| ***P53***(b) (P04637) | Growth rate assay in human cells | DMS_b | 39.37 | (Kotler *et al*, 2018) |
| ***PDE3A*** (Q14432) | DNMDP sensitivity in a glioblastoma cell line | DMS_DNMDP_100 | 36.41 | (Garvie *et al*, 2021) |
| ***SNCA*** (P37840) | Yeast growth rate hindered by toxic aggregates (reverse survival) | DMS_Miconazole | 97.26 | (Newberry *et al*, 2020) |
| ***CCR5*** (P51681) | Antibody binding | DMS_Ab2D7 | 97.97 | (Heredia *et al*, 2018) |
| ***CXCR4*** (P61073) | Surface expression levels in human cells | DMS_expression | 99.36 |  |
| ***TPMT*** (P51580) | Protein stability assessed by FACs (VAMP-seq) | DMS | 79.25 | (Matreyek *et al*, 2018) |
| ***PTEN***(a) (P60484) |  | N/A | N/A |  |
| ***PTEN***(b) (P60484) | Disruption of an artificial gene circuit in yeast | DMS_highqual_b | 85.73 | (Mighell *et al*, 2018) |
| ***VKORC1*** (Q9BQB6) | Protein stability assessed by FACs (VAMP-seq) | DMS_VAMP | 87.02 | (Chiasson *et al*, 2020) |
| ***HRAS*** (P01112) | Yeast two-hybrid | DMS_g12v | 87.30 | (Bandaru *et al*, 2017) |
| ***ADRB2*** (P07550) | Reporter expression | DMS_0.65 | 99.40 | (Jones *et al*, 2020) |
| ***CASP3*** (P42574) | Apoptotic activity assessed by a microfluidic system. | DMS | 28.63 | (Roychowdhury & Romero, 2022) |
| ***CASP7*** (P55210) |  | DMS | 29.17 |  |
| ***CYP2C9*** (P11712) | Activity profiling by click-seq | DMS_activity | 65.97 | (Amorosi *et al*, 2021) |

Table EV1 - **Summary of all DMS studies used to benchmark VEPs**.

All DMS studies that were used to benchmark VEPs including a brief description of the functional assay used to assess variant fitness. In most cases, low coverage (>40%) indicates that the study focussed on SNVs rather than all possible amino acid substitutions. For *BRCA1*, the low coverage is due to only one protein domain being assessed. Most DMS studies provided multiple sets of scores representing different assay conditions, repeats or methodologies. A single score was selected to represent each study based on median correlation against all VEPs, show in the “Selected Score” column. A full list of these assays is available in Table EV2. Where the coverage and selected score are “N/A”, a different generated by a different group was selected to represent that protein in the DMS benchmark.
