# Supplementary material for: Updated benchmarking of variant effect predictors using deep mutational scanning
Source: Mol Syst Biol. 2023 Jun 13;19(8):e11474. doi: 10.15252/msb.202211474 (PMC10407742; doi:10.15252/msb.202211474)
Supplement: Supplementary file 13 [file MSB-19-e11474-s001.pdf]

molecular systems biology

Updated benchmarking of variant effect predictors using deep mutational scanning

Benjamin Livesey and Joseph Marsh
DOI: 10.15252/msb.202211474

Corresponding author(s): Joseph Marsh (joseph.marsh@ed.ac.uk)

Review Timeline:

Submission Date:	23rd Nov 22
Editorial Decision:	16th Jan 23
Revision Received:	6th Apr 23
Editorial Decision:	3rd May 23
Revision Received:	30th May 23
Accepted:	2nd Jun 23

Editor: Maria Polychronidou

Transaction Report:

(Note: With the exception of the correction of typographical or spelling errors that could be a source of ambiguity, letters and reports are not edited. Depending on transfer agreements, referee reports obtained elsewhere may or may not be included in this compilation. Referee reports are anonymous unless the Referee chooses to sign their reports.)

16th Jan 2023

Manuscript Number: MSB-2022-11474
Title: Updated benchmarking of variant effect predictors using deep mutational scanning

Dear Dr. Marsh,

Thank you again for submitting your work to Molecular Systems Biology. We have now heard back from the three reviewers who agreed to evaluate your study. As you will see below, the reviewers appreciate that the study seems relevant for the field. However, they raise a series of concerns, which we would ask you to address in a revision.

I think that the reviewers' recommendations are rather clear and I therefore see no need to repeat any of the comments listed below. All issues raised by the referees would need to be satisfactorily addressed. Please let me know in case you would like to discuss in further detail any of the issues raised, I would be happy to schedule a call.

On a more editorial level, we would ask you to address the following points:

- Please provide a .doc version of the manuscript text (including legends for the main figures) and individual production quality figure files for the main Figures (one file per figure).

- Please provide 5 keywords.

- We have replaced Supplementary Information by the Expanded View (EV format). In this case, all additional figures can be provided as EV Figures, labelled and called out as Figure EV1, Figure EV2 etc. For detailed instructions regarding expanded view please refer to our Author Guidelines: .

- Tables S1-S9 should be provided as EV Tables (if that are relatively simple/short tables) or EV Datasets (if they are longer and more complex). Please provide one file per EV Dataset/Table. They should be labeled and called out as Table EV1, Dataset EV1 etc. For .xls files: please include in each .xls file a description of the table/dataset in a separate tab.

- Please provide a "standfirst text" summarizing the study in one or two sentences (approximately 250 characters), three to four "bullet points" highlighting the main findings and a "synopsis image" (550px width and max 400px height, jpeg format) to highlight the paper on our homepage.

- All Materials and Methods need to be described in the main text. We would encourage you to use 'Structured Methods', our new Materials and Methods format. According to this format, the Material and Methods section should include a Reagents and Tools Table (listing key reagents, experimental models, software and relevant equipment and including their sources and relevant identifiers) followed by a Methods and Protocols section in which we encourage the authors to describe their methods using a step-by-step protocol format with bullet points, to facilitate the adoption of the methodologies across labs. More information on how to adhere to this format as well as downloadable templates (.doc or .xls) for the Reagents and Tools Table can be found in our author guidelines: . An example of a Method paper with Structured Methods can be found here:

- Please include a "Disclosure Statement & Competing Interests" in the main text.

- Please include a Data availability section describing how the data, code etc. have been made available. This section needs to be formatted according to the example below:
The datasets and computer code produced in this study are available in the following databases:
- Chip-Seq data: Gene Expression Omnibus GSE46748 (https://www.ncbi.nlm.nih.gov/geo/query/acc.cgi?acc=GSE46748)
- Modeling computer scripts: GitHub (https://github.com/SysBioChalmers/GECKO/releases/tag/v1.0)
- [data type]: [full name of the resource] [accession number/identifier] ([doi or URL or identifiers.org/DATABASE:ACCESSION])

- The References should be formatted according to the Molecular Systems Biology reference style.

- Molecular Systems Biology supports formal data citations in the Reference list, to cite previously published datasets. In addition to citing the original papers that reported the data, we encourage you to also cite the relevant datasets directly in the Reference list. In the text, references to datasets are included as "Data ref: Smith et al, 2001" or "Data ref: NCBI Sequence Read Archive PRJNA342805, 2017". In the Reference list, data citations are very similar to normal literature references but must be labeled with "[DATASET]" at the end of the reference. For detailed instructions please refer to our Author Guidelines .

- For data quantification: please specify the name of the statistical test used to generate error bars and P values, the number (n) of independent experiments (specify technical or biological replicates) underlying each data point and the test used to calculate p-values in each figure legend. The figure legends should contain a basic description of n, P and the test applied. Graphs must

include a description of the bars and the error bars (s.d., s.e.m.).

- When you resubmit your manuscript, please download our CHECKLIST (https://bit.ly/EMBOPressAuthorChecklist) and include the completed form in your submission.
Please note that the Author Checklist will be published alongside the paper as part of the transparent process (https://www.embopress.org/page/journal/17444292/authorguide#transparentprocess).

If you feel you can satisfactorily deal with these points and those listed by the referees, you may wish to submit a revised version of your manuscript. Please attach a covering letter giving details of the way in which you have handled each of the points raised by the referees. A revised manuscript will be once again subject to review and you probably understand that we can give you no guarantee at this stage that the eventual outcome will be favorable.

Kind regards,

Maria

Maria Polychronidou, PhD
Senior Editor
Molecular Systems Biology

We realize that it is difficult to revise to a specific deadline. In the interest of protecting the conceptual advance provided by the work, we recommend a revision within 3 months (16th Apr 2023). Please discuss the revision progress ahead of this time with the editor if you require more time to complete the revisions. Use the link below to submit your revision:

https://msb.msubmit.net/cgi-bin/main.plex

IMPORTANT: When you send your revision, we will require the following items:
1. the manuscript text in LaTeX, RTF or MS Word format
2. a letter with a detailed description of the changes made in response to the referees. Please specify clearly the exact places in the text (pages and paragraphs) where each change has been made in response to each specific comment given
3. three to four 'bullet points' highlighting the main findings of your study
4. a short 'blurb' text summarizing in two sentences the study (max. 250 characters)
5. a 'thumbnail image' (550px width and max 400px height, Illustrator, PowerPoint or jpeg format), which can be used as 'visual title' for the synopsis section of your paper.
6. Please include an author contributions statement after the Acknowledgements section (see https://www.embopress.org/page/journal/17444292/authorguide)
7. Please complete the CHECKLIST available at (https://bit.ly/EMBOPressAuthorChecklist).
Please note that the Author Checklist will be published alongside the paper as part of the transparent process (https://www.embopress.org/page/journal/17444292/authorguide#transparentprocess).
8. When assembling figures, please refer to our figure preparation guideline in order to ensure proper formatting and readability in print as well as on screen:
https://bit.ly/EMBOPressFigurePreparationGuideline
See also figure legend guidelines: https://www.embopress.org/page/journal/17444292/authorguide#figureformat
9. Please note that corresponding authors are required to supply an ORCID ID for their name upon submission of a revised manuscript (EMBO Press signed a joint statement to encourage ORCID adoption).
(https://www.embopress.org/page/journal/17444292/authorguide#editorialprocess)
Currently, our records indicate that the ORCID for your account is 0000-0003-4132-0628.

Please click the link below to modify this ORCID:
Link Not Available

10. At EMBO Press we ask authors to provide source data for the main manuscript figures. Our source data coordinator will contact you to discuss which figure panels we would need source data for and will also provide you with helpful tips on how to upload and organize the files.

The system will prompt you to fill in your funding and payment information. This will allow Wiley to send you a quote for the article processing charge (APC) in case of acceptance. This quote takes into account any reduction or fee waivers that you may be eligible for. Authors do not need to pay any fees before their manuscript is accepted and transferred to the publisher.

EMBO Press participates in many Publish and Read agreements that allow authors to publish Open Access with reduced/no publication charges. Check your eligibility: https://authorservices.wiley.com/author-resources/Journal-Authors/open-access/affiliation-policies-payments/index.html

As a matter of course, please make sure that you have correctly followed the instructions for authors as given on the submission website.

*** PLEASE NOTE *** As part of the EMBO Press transparent editorial process initiative (see our Editorial at https://dx.doi.org/10.1038/msb.2010.72), Molecular Systems Biology publishes online a Review Process File with each accepted manuscripts. This file will be published in conjunction with your paper and will include the anonymous referee reports, your point-by-point response and all pertinent correspondence relating to the manuscript. If you do NOT want this File to be published, please inform the editorial office at msb@embo.org within 14 days upon receipt of the present letter.

Reviewer #1:

Summary
In the "Updated benchmarking of variant effect predictors using deep mutational scanning" manuscript, the authors describe their efforts to evaluate variant effect predictors (VEPs) while avoiding circularity. To achieve this, they use variant effect maps produced via deep mutational scanning as independent reference sets. Using pairwise-comparisons they produce a ranking of VEPs. For the subset of unsupervised VEPs they also perform a second evaluation using known pathogenic and benign variants from Clinvar, HGMD and gnomAD and perform a similar pairwise ranking. Finally, the authors show that both rankings show very similar outcomes.

General remarks
The manuscript is generally well-written. While each individual evaluation method employed is subject to some limitations, the authors discuss these limitations well and make good use of the fact the two methods complement each other, thus lending each other support.

The comparison includes many new VEPs some of which have not previously been independently evaluated, which will be of high interest for the clinical variant interpretation community, and more generally for geneticists and those studying protein-structure-function relationships.

Major points
-Both rankings calculated in the manuscript rely on pairwise scores, the numerical order of which is used to establish a hierarchy. However this glosses over the fact that not all of these score differences may be statistically significant. It may thus be more fair to treat statistically indistinguishable scores as a tie (i.e. as a shared rank). Especially at the top end of the hierarchy, where scores appear to be very similar, it may turn out to be that the top contenders share joint ranks.

-As the authors correctly point out, the use of ROCs can lead to overestimation of performance when class sizes are unbalanced. While they acknowledge PRCs as an alternative they only provide AUPRC metrics in the supplement and do not use them for the final ranking, pointing to the fact the PRCs are not easy to compare between proteins due to the effects of sample bias. However, this could be easily overcome using prior-balancing, such as demonstrated in the VARITY publication (Wu et al AJHG 2021)

-The reference sets from Clinvar, HGMD and gnomAD used to establish the second ranking of unsupervised VEPs merits a more careful filtering approach. A great example of this can be seen in Pejaver et al. AJHG 2022. This includes filtering for variant call quality, sample size and allele frequency in gnomAD and for quality rating in Clinvar.

-Re: "We improved upon our previous VEP rank score calculations by performing a comparison between all pairs of VEPs using the Spearman's correlation between each VEP and DMS data across only variants for which both VEPs produced predictions." This does indeed seem like an improvement, but doesn't resolve all issues: imagine two equally-good VEPs that each have a distinct (but equally sized) fraction of variants for which performance is subpar. If one of these VEPs chooses not to report predictions for their subpar space, while the other does, then the first will be judged the winner. Furthermore, VEPs that report scores for a smaller fraction of variants should be penalized, or perhaps coverage should be another performance measure.

Minor points
-A key point in the manuscript is the fact that the two ranking systems the authors employed complement each other with respect to their strengths and weaknesses. The authors may want emphasize this point earlier on as an important selling point (especially in the abstract). As it is, readers will spend some time being dubious as to the validity of each method until this point is made near the end of the paper.

-The introduction mentions the challenge that rarity poses in classifying pathogenic variants, but might also mention that it also poses a challenge for classifying rare benign variants.

-Results section mentions what the numbers of DMS and VEPs have increased to, but should list what they were before. The strategy used where multiple DMS datasets were available is reasonable. The authors might try (or at least discuss the possibility of) filtering out regions of DMS maps where scores correlate poorly with VEPs in general. This might have made an impact for MTHFR, where the authors treated the regulatory domain separately.

-The authors briefly discuss the reasons why some DMS maps may correlate less with the VEPs tested, including the model organism and type of assay used in the DMS experiment, but another reason may be the mode of inheritance underlying the genotype-phenotype relationship. For example, SNCA is typically dominant and can harbour pathogenic variants that are both loss of function and gain of function. The latter are less likely to be predicted well by VEPs.

-Another example of the potential impact of mode of inheritance can be found in calmodulin, in which pathogenic variants are likely to be dominant-negative, so that variants causing intermediate impacts on protein function may cause disease while other variants which completely disrupt protein folding may be tolerated as they can no longer disrupt protein complexes. The DMS assay was carried out in a haploid context, so that it measures loss of function but did not specifically measure the types of loss of function that yield dominant negative effects on wild-type calmodulin. Of course, VEPs are similarly unaware that intermediate variant effects are more likely to be pathogenic than profound null effects, so that despite the positive correlation of VEP and DMS, neither may be monotonically related to pathogenicity.

-In the Meier et al paper, there were multiple versions of ESM-1v, one of which was trained (or hyper-parameter-tuned at any rate) using DMS data. I think the use of zero-shot ESM-1v means that the scores are clean, but would be good to confirm explicitly that the ESM-1v scores used here were not informed by DMS.

Reviewer #2:

In this study, Livesey and Marsh provide an update to their previous benchmarking of variant effects predictors (VEPs). They assess the performance of VEPs at predicting the functional effects of mutations (as measured by deep mutational scanning experiments) as well as their clinical significance (as reported in ClinVar). They pay a special attention at biases coming from data circularity and distinguish supervised from unsupervised VEPs. Following the ranking of VEPs at these tasks, the authors conclude that some of the new unsupervised methods, which are less affected by data circularity, perform best.

Predicting the impact of genetic variation on gene function and its phenotypic consequences is one of the major goals of genetics. With the recent developments of deep mutational scanning assays and deep learning, this goal now appears within reach. As a result, this field is rapidly growing and the number and diversity of VEPs is increasing fast. It thus becomes harder for non-experts to choose an appropriate VEP for their prediction task. A robust and regular benchmarking of VEPs performance is thus important not only to identify best performers for immediate use, but also to monitor progress and identify approaches with higher potential in order to guide future development. In this respect, the work done by Livesey and Marsh will be of high value for the deep mutational scanning field and the clinical genetics community at large. The work done is robust, with special attention to biases as mentioned above and I do not see any technical shortcoming. I therefore support publication in Molecular Systems Biology with only a few very minor revisions.

- In addition to comparing the performance of VEPs on DMS data using the rank score in figure 2, it would also be informative to show the distribution of correlation coefficient as in figure 1, but with VEPs on the x-axis and DMS datasets as data points. In my opinion this gives a better idea of the overall performance compared to the rank score that can suffer from a sort of thresholding effect when selecting a "winner".
- Figure 4, the median line is black, not orange as stated in the legend

Reviewer #3:

In this manuscript, B.J. Livesey and J.A. Marsh provide an update to their pathogenicity prediction benchmarking study published in Molecular Systems Biology (2020)16:e9380. They increased the number of Deep Mutational Scanning (DMS) data sets and included several more variant effect predictors (VEPs) in their benchmarks. Most of their conclusions are based on the benchmark on the DMS data, however a subset of VEPs (which the authors identify as "unsupervised") is also evaluated on a ClinVar benign vs. pathogenic set. The manuscript touches on the discussion whether DMS data, or only certain types of DMS experiments, can approximate disease phenotypes, allowing them to be used as a proxy for pathogenic vs. benign. There is also

some discussion about the limitations of using ClinVar benchmarks due to overlaps with training data sets. I generally agree with most statements and conclusions presented in the manuscript but have some issues with the order and clarity in which they are presented.
Specifically for the order, the authors seem to make broad and general statements first, which they later put into perspective or weaken. I do not believe this is a good way of communicating results.

Here my major points:

- DMS as a proxy for pathogenicity: It is important to be completely upfront that DMS does not measure organismal pathogenicity, but specific molecular functions or cellular viability/growth. Statements like "One must ensure that the fitness metric being measured is relevant to human pathogenic conditions" come too late. Also, when considering multiple measures for the same protein, some might be more related to pathogenicity than others. [As applied by the authors, using a median over existing VEPs to decide which measure correlates the best with organismal pathogenicity seems a very reasonable approach.]

- Exclusion of supervised approaches: This is an oversimplification. Approaches might be supervised/use labeled data, but this data might not overlap pathogenic variant sets used in model validation/assessment. The point that is discussed for Eigen does not only apply to Eigen, but also to CADD and DANN (all using the same training data). CADD (2014 publication) highlights in the supplement though that including PolyPhen-2 does not result in a relevant bleed-over effect and that if PolyPhen is removed from model training, it can be compensated by the individual coefficients for amino acid substitutions. Thus, the argument must be based on the training objective rather than the inclusion of individual features in these high-feature models. I would also like to note that there are two PolyPhen models (HumDiv and HumVar). Only HumDiv models should be showing ClinVar training overlap. I therefore encourage the authors to include all methods in their benchmarks and to rather have a different color-coding for those that are trained on ClinVar, those that might have some information derived from known pathogenic variation and those that could be considered free of such effects.

- "unsupervised VEP predictions cannot be overtly influenced in the same way, as these methods are not trained using labelled data." -- This is not true as unsupervised methods are directly influenced by biases in the underlying data set.

- "VARITY_R and VARITY_ER retain 4th and 5th ranked places respectively, and the rank scores even improve marginally (Table S7). Thus, the strong performance of VARITY does not appear to be due to data circularity." -- Learning DMS performance might still be something else than pathogenicity and the model learned DMS performance.

- "DMS data are a valid representation of likely predictor performance against clinically relevant human missense variants." -- One of the statements that needs to be weakened right away, rather than ending the results section with it.

Minor points:

- "Other common DMS approaches such as measuring protein expression levels by VAMP-seq (Matreyek et al, 2018) or cell-surface expression, or measuring specific protein interaction affinities tended to be less correlated with VEP predictions or produced mixed results." -- These are also expected to provide less information about maintaining the proteins' function and pathogenicity.
- "while putatively benign variants were obtained from gnomAD (Karczewski et al, 2020), excluding those that were also present in the pathogenic set." -- provide AF threshold in text
- "It is likely that the ClinVar and gnomAD databases are not suitable for identifying such pharmacogenetic variants, as they would appear benign until a patient was exposed to the drug. Furthermore," -- Not sure this sentence makes sense. The reason they are in ClinVar is patients that have pharmacogenetic problems, isn't it? GnomAD just catalogs variants observed in individuals free of early-on-set disease.

- "it is likely that a large number of these variants would be present in gnomAD, thus greatly reducing the apparent predictive performance of all methods for this protein. For several proteins, our pathogenic and putatively benign samples are highly unbalanced" -- Is this recognizing the varying effect size? I.e., pathogenicity being on a continuous scale (low for immune/pharmacogenetic variants; high for early onset deadly diseases?), but the different DMS all being on different scales? This could be much clearer.

- "For several proteins, our pathogenic and putatively benign samples are highly unbalanced (Table S8), which can potentially lead to ROC AUCs being an overly optimistic assessment of performance. The alternative is to use precision-recall curves, which focus on correct prediction of the positive (pathogenic) class but are sensitive to sample balance, making comparisons between proteins difficult." -- Another alternative is to sample... Overrepresentation of certain proteins should be addressed by downsampling and, similarly, the number of pathogenic and benign variants should be controlled on a protein level.

- "It has been noted that nucleotide-based alignments (such as those that form the basis of GERP++, SiPhy and PhyloP) are considerably noisier than protein alignments (Wernersson & Pedersen, 2003) and that protein-based alignments allow for more distantly related sequences to be included in the alignment (Pearson, 2013)." -- Reference from 2003 seems very dated here, we did not even have an appreciable number of genomic sequences then and alignment approaches have improved. Is there a

more up-to-date reference for that? Similarly, the question for the 2013 reference and whether it still holds (e.g., https://www.nature.com/articles/s41586-020-2876-6).

Response to reviewers

Thank you for taking time to review our manuscript. The following points detail the steps we have taken to address all the concerns raised by the reviewers.

Response to the points raised by reviewer 1

-Both rankings calculated in the manuscript rely on pairwise scores, the numerical order of which is used to establish a hierarchy. However this glosses over the fact that not all of these score differences may be statistically significant. It may thus be more fair to treat statistically indistinguishable scores as a tie (i.e. as a shared rank). Especially at the top end of the hierarchy, where scores appear to be very similar, it may turn out to be that the top contenders share joint ranks.

In order the gauge the statistical significance of our findings we implemented a bootstrapping approach whereby the ranking calculations were repeated 1000 times with data sampled with replacement. The matrices generated by this method are available as Table EV7. The findings indicate that the top several VEPs cannot be said to be statistically better than one another (particularly in the second benchmark), and the text now reflects these findings. We found that ESM-1v was not significantly higher ranked than EVE (p=0.130), nor was EVE significantly higher ranked than DeepSequence (p=0.123) or VARITY_R (p=0.062). We have also used the data to add standard deviation error bars to Figure 2.

-As the authors correctly point out, the use of ROCs can lead to apparent overestimation of performance when class sizes are unbalanced. While they acknowledge PRCs as an alternative they only provide AUPRC metrics in the supplement and do not use them for the final ranking, pointing to the fact the PRCs are not easy to compare between proteins due to the effects of sample bias. However, this could be easily overcome using prior-balancing, such as demonstrated in the VARITY publication (Wu et al AJHG 2021)

We have implemented prior balancing for calculation of the balanced precision-recall AUC as described in the VARITY paper and used it to complement our analysis with AUROC. We retained the use of AUROC in the main analysis but also performed the calculations of main ROC analyses with AUBPRC as well (Fig EV3 and EV4). Overall, both approaches give very similar rankings. The text of the final two results sections have been updated and the balanced PR calculations have been added to the methods section.

-The reference sets from Clinvar, HGMD and gnomAD used to establish the second ranking of unsupervised VEPs merits a more careful filtering approach. A great example of this can be seen in Pejaver et al. AJHG 2022. This includes filtering for variant call quality, sample size and allele frequency in gnomAD and for quality rating in Clinvar.

We implemented filtering of ClinVar variants to remove any 0* variants from the benchmarking dataset. We also manually curated the HGMD variants to remove any not directly associated with a pathogenic condition e.g. drug response or altered function. It was not possible to filter the gnomAD variants by allele frequency as doing so according to the method of Pejaver et al. left less than 10 variants for all proteins except *P53*, *MSH2* and *BRCA1* (even while filtering with a cutoff of 0.001). We ensured that all gnomAD variants passed the gnomAD internal filter (At least on sample with a high-quality genotype (depth >=10, quality >= 20, minor allele balance >0.2)). The first paragraph of the "Performance of DMS compared against datasets of pathogenic and benign missense variants" section now discusses the

quality of gnomAD and the issues with filtering. The Methods section now describes the filtering details for ClinVar and the gnomAD internal filtering criteria.

-Re: "We improved upon our previous VEP rank score calculations by performing a comparison between all pairs of VEPs using the Spearman's correlation between each VEP and DMS data across only variants for which both VEPs produced predictions." This does indeed seem like an improvement, but doesn't resolve all issues: imagine two equally-good VEPs that each have a distinct (but equally sized) fraction of variants for which performance is subpar. If one of these VEPs chooses not to report predictions for their subpar space, while the other does, then the first will be judged the winner. Furthermore, VEPs that report scores for a smaller fraction of variants should be penalized, or perhaps coverage should be another performance measure.

While the concern is certainly justified, all the VEPs we have assessed have at least 64.5% of the protein covered while most have over 95%. To explore the possibility that low-coverage predictors may overperform, we performed the ranking analysis using only mutations possible by a SNV (so that nucleotide-level predictors don't get unfairly penalised) and filled in the remaining missing predictions with the predictor's most 'benign' result (Table EV9). Overall, the ranking changes are minor, but the results do appear to indicate that mutationTCN was overperforming as it dropped from 11th to 22nd.

-A key point in the manuscript is the fact that the two ranking systems the authors employed complement each other with respect to their strengths and weaknesses. The authors may want emphasize this point earlier on as an important selling point (especially in the abstract). As it is, readers will spend some time being dubious as to the validity of each method until this point is made near the end of the paper.

The point that the two rankings are intended to be complementary to one another has now been made stronger in the abstract and in the final paragraph of the introduction.

-The introduction mentions the challenge that rarity poses in classifying pathogenic variants, but might also mention that it also poses a challenge for classifying rare benign variants.

The introduction now also mentions rare benign variants as well (In fact, benign variants may be an even greater challenge!)

-Results section mentions what the numbers of DMS and VEPs have increased to, but should list what they were before.

The first paragraph of the results section has been updated to give the VEP and DMS count in this study and the previous study.

-The strategy used where multiple DMS datasets were available is reasonable. The authors might try (or at least discuss the possibility of) filtering out regions of DMS maps where scores correlate poorly with VEPs in general. This might have made an impact for MTHFR, where the authors treated the regulatory domain separately.

To address this, we used a scanning window (20 amino acids) to calculated mean VEP correlation along the length of each protein. We then discarded the central 10 amino acids from the window if the correlation was under 1 standard deviation from the mean (the start / end 10 amino acids were also

discarded if the first or last window fell under the threshold). The correlation analysis was repeated with the remaining data (Table EV10). We found only minor changes in performance, even for *MTHFR*.

-The authors briefly discuss the reasons why some DMS maps may correlate less with the VEPs tested, including the model organism and type of assay used in the DMS experiment, but another reason may be the mode of inheritance underlying the genotype-phenotype relationship. For example, SNCA is typically dominant and can harbour pathogenic variants that are both loss of function and gain of function. The latter are less likely to be predicted well by VEPs.

We have added a paragraph to the Discussion talking about the difficulty that VEPs have in predicting the effects of non-LOF mutations. We used specific examples in SNCA (gain-of-function) and CALM1 (dominant negative).

Another example of the potential impact of mode of inheritance can be found in calmodulin, in which pathogenic variants are likely to be dominant-negative, so that variants causing intermediate impacts on protein function may cause disease while other variants which completely disrupt protein folding may be tolerated as they can no longer disrupt protein complexes. The DMS assay was carried out in a haploid context, so that it measures loss of function but did not specifically measure the types of loss of function that yield dominant negative effects on wild-type calmodulin. Of course, VEPs are similarly unaware that intermediate variant effects are more likely to be pathogenic than profound null effects, so that despite the positive correlation of VEP and DMS, neither may be monotonically related to pathogenicity.

We have added a further paragraph to the Discussion addressing this scenario.

-In the Meier et al paper, there were multiple versions of ESM-1v, one of which was trained (or hyper-parameter-tuned at any rate) using DMS data. I think the use of zero-shot ESM-1v means that the scores are clean, but would be good to confirm explicitly that the ESM-1v scores used here were not informed by DMS.

We have made it clear in Table 2 and the "Benchmarking of VEPs using DMS data" section that ESM-1v was used without any additional training or tuning.

Response to reviewer 2

-In addition to comparing the performance of VEPs on DMS data using the rank score in figure 2, it would also be informative to show the distribution of correlation coefficient as in figure 1, but with VEPs on the x-axis and DMS datasets as data points. In my opinion this gives a better idea of the overall performance compared to the rank score that can suffer from a sort of thresholding effect when selecting a "winner".

We have added the suggested figure as Figure EV1 in the format of a bar chart (a scatter plot was too messy). In addition, as per reviewer 1's comments we have also introduced a significance metric for both ranking systems to show how statistically significant the ranked positions of each predictor are.

-Figure 4, the median line is black, not orange as stated in the legend

The legend has been fixed!

Response to reviewer 3

-DMS as a proxy for pathogenicity: It is important to be completely upfront that DMS does not measure organismal pathogenicity, but specific molecular functions or cellular viability/growth. Statements like "One must ensure that the fitness metric being measured is relevant to human pathogenic conditions" come too late. Also, when considering multiple measures for the same protein, some might be more related to pathogenicity than others. [As applied by the authors, using a median over existing VEPs to decide which measure correlates the best with organismal pathogenicity seems a very reasonable approach.]

The statements regarding the limitations of DMS have been significantly expanded on in the final paragraph of the introduction.

"While benchmarking VEPs against DMS datasets greatly mitigates the issue of data circularity, the relevance of such datasets to human pathogenic conditions may be more circumspect. For example, in the case of the dominant-negative effect, one would expect mutations with a mild effect on individual protein function to cause a more severe phenotype than highly destabilising mutations. Other factors such as limitations of the experimental system and relevance of the functional assay to disease mechanisms can also affect the usefulness of such datasets."

-Exclusion of supervised approaches: This is an oversimplification. Approaches might be supervised/use labeled data, but this data might not overlap pathogenic variant sets used in model validation/assessment. The point that is discussed for Eigen does not only apply to Eigen, but also to CADD and DANN (all using the same training data). CADD (2014 publication) highlights in the supplement though that including PolyPhen-2 does not result in a relevant bleed-over effect and that if PolyPhen is removed from model training, it can be compensated by the individual coefficients for amino acid substitutions. Thus, the argument must be based on the training objective rather than the inclusion of individual features in these high-feature models. I would also like to note that there are two PolyPhen models (HumDiv and HumVar). Only HumDiv models should be showing ClinVar training overlap. I therefore encourage the authors to include all methods in their benchmarks and to rather have a different color-coding for those that are trained on ClinVar, those that might have some information derived from known pathogenic variation and those that could be considered free of such effects.

While the exclusion of Eigen may appear to be overkill, we are striving to maintain a consistent system that minimizes any possibility of data circularity from contaminating the results. This applies not only to ClinVar and HGMD variants but also to the gnomAD variants that make up the majority of the putatively benign dataset. Regarding HumVar specifically, it was assembled from human sequences in UniProt with pathogenic or neutral annotations. It should overlap considerably at both with gnomAD and ClinVar. We have added the supervised VEPs as supplemental figures (EV5 for the clinical data benchmark and EV6 for the comparison between the benchmarks) and discussed the findings in the relevant sections.

-"unsupervised VEP predictions cannot be overtly influenced in the same way, as these methods are not trained using labelled data." -- This is not true as unsupervised methods are directly influenced by biases in the underlying data set.

Clarification has been added to the statement:

"The issues of type 1 and 2 data circularity apply primarily to supervised VEPs; in contrast, unsupervised VEP predictions cannot be overtly influenced in the same way, as these methods are not trained using

labelled data although biases may still emerge based on the composition of the underlying data (often a MSA). It is still possible that some unsupervised VEPs are tweaked based on performance against clinical observations that could re-introduce type 1 circularity into performance assessments but in general, we consider unsupervised VEPs immune to these forms of bias."

-"VARITY_R and VARITY_ER retain 4th and 5th ranked places respectively, and the rank scores even improve marginally (Table S7). Thus, the strong performance of VARITY does not appear to be due to data circularity." -- Learning DMS performance might still be something else than pathogenicity and the model learned DMS performance.

We have added a disclaimer that VARITY may have learned some underlying general features of DMS which could also explain its consistent performance:

"Thus, the strong performance of VARITY does not appear to be due to data circularity although the possibility also exists that VARITY has learned to predict some features of DMS in general."

-"DMS data are a valid representation of likely predictor performance against clinically relevant human missense variants." -- One of the statements that needs to be weakened right away, rather than ending the results section with it.

This statement has been removed from the end of the Results section. Discussion on the usefulness of DMS for benchmarking has been confined to the Discussion section.

-"Other common DMS approaches such as measuring protein expression levels by VAMP-seq (Matreyek et al, 2018) or cell-surface expression, or measuring specific protein interaction affinities tended to be less correlated with VEP predictions or produced mixed results." -- These are also expected to provide less information about maintaining the proteins' function and pathogenicity.

We have added caveats regarding the fitness measurements returned by VAMP-seq and other expression-quantifying DMS methodologies:

"This is likely due to a disconnect between the specific fitness definition of the assay and the more general fitness effects predicted by VEPs. VAMP-seq, for example only identifies variants that negatively affect protein stability as low fitness, while the protein itself may be non-functional but stable."

-"while putatively benign variants were obtained from gnomAD (Karczewski et al, 2020), excluding those that were also present in the pathogenic set." -- provide AF threshold in text

Applying an allele frequency threshold was not possible due to the small number of variants remaining (for all genes but P53, BRCA1 and MSH2). All gnomAD variants pass the internal gnomAD quality check (At least on samples with a high-quality genotype (depth >=10, quality >= 20, minor allele balance >0.2) and all ClinVar variants have a review status of at least one star. We have added gnomAD quality filter description to methods, and mentioned the issues with filtering gnomAD in the main text.

-"It is likely that the ClinVar and gnomAD databases are not suitable for identifying such pharmacogenetic variants, as they would appear benign until a patient was exposed to the drug. Furthermore," -- Not sure this sentence makes sense. The reason they are in ClinVar is patients that have pharmacogenetic problems, isn't it? GnomAD just catalogs variants observed in individuals free of early-on-set disease.

This was an extremely helpful comment! CYP2C9 and CCR5 have been removed from the set of proteins used for the second benchmark as the phenotypic outcome of mutants in these proteins were not *truly* pathogenic. Discussed this point in the main text:

“While both CYP2C9 and CCR5 had enough pathogenic variants to be included in this analysis, close inspection indicated that most of the HGMD variants were not, in fact truly pathogenic. CYP2C9 is an enzyme involved in drug metabolism, and most variants in ClinVar and HGMD have an altered drug response phenotype. Using these variants as a “pathogenic” dataset for the purpose of calculating AUBPRC produces extremely poor results across all VEPs and DMS datasets (Fig EV2a). Another contributing factor is likely the presence of many drug response variants in gnomAD which would not be filtered out. Using a specialised database such as PharmVar (Gaedigk et al, 2018) may be more appropriate for assessing the performance of VEPs and DMS datasets for variant interpretation in this protein. CCR5 is a cell-surface chemokine receptor expressed by T-cells and macrophages. The protein is also important for HIV cell entry, and most ClinVar and HGMD entries are variants that alter HIV binding affinity. While AUBPRC results are not particularly poor when labelling these variants as pathogenic (Fig EV2b), variants that affect HIV entry are not necessarily relevant to human genetic disease.”

We also created a supplementary figure (EV2) demonstrating how poorly CYP2C9 variants perform when used directly for variant classification, and manually curated all HGMD variants to remove further variants with no direct disease association.

-"For several proteins, our pathogenic and putatively benign samples are highly unbalanced (Table S8), which can potentially lead to ROC AUCs being an overly optimistic assessment of performance. The alternative is to use precision-recall curves, which focus on correct prediction of the positive (pathogenic) class but are sensitive to sample balance, making comparisons between proteins difficult." -- Another alternative is to sample... Overrepresentation of certain proteins should be addressed by downsampling and, similarly, the number of pathogenic and benign variants should be controlled on a protein level.

Reviewer 1 provided an alternative solution, balanced area under the precision-recall curve, which we have now adopted. The results of using this alternate assessment metric are shown in Figures EV3 and EV4.

-"It has been noted that nucleotide-based alignments (such as those that form the basis of GERP++, SiPhy and PhyloP) are considerably noisier than protein alignments (Wernersson & Pedersen, 2003) and that protein-based alignments allow for more distantly related sequences to be included in the alignment (Pearson, 2013)." -- Reference from 2003 seems very dated here, we did not even have an appreciable number of genomic sequences then and alignment approaches have improved. Is there a more up-to-date reference for that? Similarly, the question for the 2013 reference and whether it still holds (e.g., https://www.nature.com/articles/s41586-020-2876-6).

This is a very good point, and we could not actually find any recent references regarding the problem. One aspect is that, since many of the nucleotide-level methods are relatively old and have not been updated, they may still suffer from nucleotide alignment issues despite technology having been improved. We have updated the text to reflect our uncertainty on whether the issue still applies.

Other changes to the manuscript

In addition to changes made to reviewer's comments, we have also made several other methodological changes summarised below.

- The TPK1 DMS data predicted clinical outcome better when the scale was inverted relative to the CALM1 DMS dataset produced by the same group. We have inverted the scale of this dataset for Figure 3/EV3 as it represents the best use of the data for determining clinical outcome. This is also briefly discussed in the text in the "Performance of DMS compared to VEPs against datasets of pathogenic and benign missense variants" section of the Results.

- We also inverted the scale of Fathmm predictions used to plot Figure EV5 and Figure EV6 as Fathmm was also more predictive of clinical outcome with the inverted scale. The figure legends now note this change.

- Points for CYP2C9 and CCR5 were excluded from Figure EV7 for the same reason they were excluded from Figure 3/EV3 (lack of confidence that the pathogenic variants used to calculate AUROC are truly pathogenic).

- The VESPA predictor has been renamed "VESPAl" and the MTBAN predictor has been renamed "mutationTCN". The downloads used to obtain these scores were for 'light' versions of the predictors, not the full predictors.

3rd May 2023

Manuscript Number: MSB-2022-11474R
Title: Updated benchmarking of variant effect predictors using deep mutational scanning

Dear Dr. Marsh,

Thank you for sending us your revised manuscript. We have now heard back from the two reviewers who were asked to evaluate your revised study. As you will see below, the reviewers think that the study has improved as a result of the performed revisions. However, they still raise some remaining concerns, which we would ask you to address in a final round of revision. We would also ask you to address a few minor editorial issues listed below.

- Panels A and B should be labelled on Figure EV5.

- Please include callouts for Fig. EV4A-B, EV5B, EV7A-B in the main text.

- Please complete the title page of the author checklist.

- Tables EV8-EV10 are rather long/complex and they should renamed to Dataset EV1-EV3. Please make sure that their callouts in the text are updated.

- The title of the "Conflict of Interest" statement should be changed to "Disclosure and Competing Interests Statement".

Please resubmit your revised manuscript online, with a covering letter listing amendments and responses to each point raised by the referees. Please resubmit the paper **within one month** and ideally as soon as possible. If we do not receive the revised manuscript within this time period, the file might be closed and any subsequent resubmission would be treated as a new manuscript. Please use the Manuscript Number (above) in all correspondence.

Click on the link below to submit your revised paper.

https://msb.msubmit.net/cgi-bin/main.plex

As a matter of course, please make sure that you have correctly followed the instructions for authors as given on the submission website.

Thank you for submitting this paper to Molecular Systems Biology.

Kind regards,

Maria

Maria Polychronidou, PhD
Senior Editor
Molecular Systems Biology

If you do choose to resubmit, please click on the link below to submit the revision online before 2nd Jun 2023.

https://msb.msubmit.net/cgi-bin/main.plex

IMPORTANT:
Please note that corresponding authors are required to supply an ORCID ID for their name upon submission of a revised manuscript (EMBO Press signed a joint statement to encourage ORCID adoption).
(https://www.embopress.org/page/journal/17444292/authorguide#editorialprocess)
Currently, our records indicate that the ORCID for your account is 0000-0003-4132-0628.

Please click the link below to modify this ORCID:
Link Not Available

The system will prompt you to fill in your funding and payment information. This will allow Wiley to send you a quote for the

article processing charge (APC) in case of acceptance. This quote takes into account any reduction or fee waivers that you may be eligible for. Authors do not need to pay any fees before their manuscript is accepted and transferred to the publisher.

*** PLEASE NOTE *** As part of the EMBO Press transparent editorial process initiative (see our Editorial at https://dx.doi.org/10.1038/msb.2010.72 , Molecular Systems Biology will publish online a Review Process File to accompany accepted manuscripts. When preparing your letter of response, please be aware that in the event of acceptance, your cover letter/point-by-point document will be included as part of this File, which will be available to the scientific community. More information about this initiative is available in our Instructions to Authors. If you have any questions about this initiative, please contact the editorial office (msb@embo.org).

Reviewer #1:

We are happy to see that the authors have addressed the points we previously raised and strengthened the manuscript. Unfortunately, in several cases the improved approaches were not applied throughout and/or relegated to the supplement instead of actually replacing the previous versions. These points also need to be better integrated into the main text and figures. More details below:

>> Both rankings calculated in the manuscript rely on pairwise scores, the numerical order of which is used to establish a hierarchy. However this glosses over the fact that not all of these score differences may be statistically significant. It may thus be more fair to treat statistically indistinguishable scores as a tie (i.e. as a shared rank). Especially at the top end of the hierarchy, where scores appear to be very similar, it may turn out to be that the top contenders share joint ranks.
>
> In order the gauge the statistical significance of our findings we implemented a bootstrapping approach whereby the ranking calculations were repeated 1000 times with data sampled with replacement. The matrices generated by this method are available as Table EV7. The findings indicate that the top several VEPs cannot be said to be statistically better than one another (particularly in the second benchmark), and the text now reflects these findings. We found that ESM-1v was not significantly higher ranked than EVE ($p=0.130$), nor was EVE significantly higher ranked than DeepSequence ($p=0.123$) or VARITY_R ($p=0.062$). We have also used the data to add standard deviation error bars to Figure 2.

It is great to see that the significance testing added value here. Unfortunately it seems that the authors only implemented these tests for the evaluation against DMS maps, but not for the evaluation against clinical variant sets.

The results for the former also need to be worked into the text better.

For example, the abstract says "The top VEPs are dominated by unsupervised methods including EVE, DeepSequence and ESM-1v, a protein language model that ranked first overall" According to the results, however, ESM-1v was only tied for top rank. As VARITY was tied for second, I suppose one could say that it was outnumbered by unsupervised methods, but perhaps only dominated by one of them?

Similarly, the flow of the results section still states that ESM1-v performed best (followed by a short explanation of how ESM1-v works), only to then state that differences were not significant, which is a bit contradictory. It may be best to lead with the fact that the top performers are statistically indistinguishable. This could also be emphasized in all figures where methods are compared, using brackets and stars to indicate which differences are significant.

>> As the authors correctly point out, the use of ROCs can lead to apparent overestimation of performance when class sizes are unbalanced. While they acknowledge PRCs as an alternative they only provide AUPRC metrics in the supplement and do not use them for the final ranking, pointing to the fact the PRCs are not easy to compare between proteins due to the effects of sample bias. However, this could be easily overcome using prior-balancing, such as demonstrated in the VARITY publication (Wu et al AJHG 2021)
>
> We have implemented prior balancing for calculation of the balanced precision-recall AUC as described in the VARITY paper and used it to complement our analysis with AUROC. We retained the use of AUROC in the main analysis but also performed the calculations of main ROC analyses with AUBPRC as well (Fig EV3 and EV4). Overall, both approaches give very similar rankings. The text of the final two results sections have been updated and the balanced PR calculations have been added to the methods section.

This is a great improvement, although the main text still primarily discusses the old AUROC metric while relegating the AUBPRC result to the supplement.

>> Re: "We improved upon our previous VEP rank score calculations by performing a comparison between all pairs of VEPs using the Spearman's correlation between each VEP and DMS data across only variants for which both VEPs produced predictions." This does indeed seem like an improvement, but doesn't resolve all issues: imagine two equally-good VEPs that

each have a distinct (but equally sized) fraction of variants for which performance is subpar. If one of these VEPs chooses not to report predictions for their subpar space, while the other does, then the first will be judged the winner. Furthermore, VEPs that report scores for a smaller fraction of variants should be penalized, or perhaps coverage should be another performance measure.
>
> While the concern is certainly justified, all the VEPs we have assessed have at least 64.5% of the protein covered while most have over 95%. To explore the possibility that low-coverage predictors may overperform, we performed the ranking analysis using only mutations possible by a SNV (so that nucleotide-level predictors don't get unfairly penalised) and filled in the remaining missing predictions with the predictor's most 'benign' result (Table EV9). Overall, the ranking changes are minor, but the results do appear to indicate that mutationTCN was overperforming as it dropped from 11 th to 22 nd .

This is another case of a new result that needs to be better integrated into the flow of the text. On page 5, the manuscript still describes the original comparison approach which rewards predictors for only reporting on a small fraction of variants instead of penalizing them. The corrected approach is only described much later on page 6. The results of this correction are also not reflected in the main figure 2 and were instead relegated to a supplementary table.

>> The strategy used where multiple DMS datasets were available is reasonable. The authors might try (or at least discuss the possibility of) filtering out regions of DMS maps where scores correlate poorly with VEPs in general. This might have made an impact for MTHFR, where the authors treated the regulatory domain separately.
>
> To address this, we used a scanning window (20 amino acids) to calculated mean VEP correlation along the length of each protein. We then discarded the central 10 amino acids from the window if the correlation was under 1 standard deviation from the mean (the start / end 10 amino acids were also discarded if the first or last window fell under the threshold). The correlation analysis was repeated with the remaining data (Table EV10). We found only minor changes in performance, even for MTHFR.

Great that the authors have investigated this.

Other issues:
"Recalculating the VEP rankings with these data (Table EV9)..." In addition to the need for significance tests noted above, there were no clearly identified ranks in this table.

Re: "The remaining data were used to re-calculate the rank scores (Table EV10).", there were no clearly identified ranks in this table either.

It appears that EV9 and EV10 were originally spreadsheets that were exported to PDF format. Unfortunately that made them hard to read due to columns being split over multiple pages, and some VEP names are cut off. If possible the authors may want to submit the original spreadsheets as supplements instead.

The points made in the context of not screening out benign variants based on allele frequency misses an opportunity to make a point made in both REVEL and VARITY papers, that common benign variants appear not to be representative of rare benign variants. Because the main value of VEPs is to make predictions for rare variants, this would argue that not only should one NOT exclude rare benign variants, there is an argument to include ONLY the subset of benign variants that are rare.

The discussion might note that an alternative 'clean' form of validation for VEPs is the use of prospective or case control cohorts, whereby methods can be ranked according to ability to predict phenotype or case vs. control status.

Reviewer #3:

In this manuscript, B.J. Livesey and J.A. Marsh provide an update to their pathogenicity prediction benchmarking study published in Molecular Systems Biology (2020)16:e9380. Compared to the earlier study, they increased the number of Deep Mutational Scanning (DMS) data sets and included several more variant effect predictors (VEPs) in their benchmarks. Most of conclusions are based on benchmarking DMS data, however evaluations are done on a ClinVar pathogenic set, too. The manuscript touches on the discussion whether DMS data, or only certain types of DMS experiments, can approximate disease phenotypes, allowing them to be used as a proxy for pathogenic vs. benign. There is also some discussion about the limitations of using ClinVar benchmarks due to overlaps with training data sets. The authors show that there is a high correlation between methods performing well on DMS and clinical variants, supporting the general idea of using DMS for VEP assessment. The manuscript is interesting and brings up some interesting discussion points that are probably still not known to a wider audience.

The authors have provided a revision to their manuscript after an initial round of reviews. Here they adjusted some statements, assessed the statistical significance of the observed performance differences and included more of the VEPs in some of their figures. Unfortunately, this revision did not include a document marked for edits (red line). I would encourage the authors to always provide such document (even if not formally requested by the journal). Similarly, and this mostly goes to the journal here, the merged document for the reviewers does not include numbers/names of the figure files. It is really difficult to match them up

given the count of main and extended view figures.

I still have some unresolved comments:

Reviewers asked about an allele frequency threshold for the benign set from gnomAD and the response of the authors is very surprising here: "Applying an allele frequency threshold was not possible due to the small number of variants remaining". Can the authors please check what is happening here? There should be sufficiently many variants in gnomAD to have a minimum allele frequency threshold for those genes. It does not make sense to use singletons from gnomAD in a benign set. - To cite the authors introduction here: " Pathogenic variants are enriched among the rarest occurring variants in the human population (Wang et al, 2021)". Typically, a frequency threshold of 1 or 5% is used when using "common" variants as benign instead of the biased set of "benign" variants from ClinVar.

Along those lines, it should be possible to match the number of common and pathogenic variants in this analysis on a gene level, rather than some overall correction for balanced AUROCs. It is important to represent genes with the same number of pathogenic and benign variants. Otherwise, you are starting to test whether scores identify genes with many (known) pathogenic variants, i.e., whether they work on a gene level and not a variant level. The intuition with which the use of AUROCs is motivated in the manuscript (i.e., that it does not matter if certain genes are better covered with pathogenic variants) is therefore not correct.

Considering the prominent discussion about assigning Eigen as a supervised method, maybe it is worthwhile to also discuss CADD, DANN, PrimateAI and probably some others, which also trained on labeled data (thus are in the supervised group), but not pathogenic/benign labels.

Typos:
- "\Introduction"
- "which makes comparisons [of] different proteins difficult"
- "from 985 protein."

We thank the reviewers for once again taking the time to look over our work and provide insightful comments. We provide a detailed response to all comments below.

Reviewer 1

It is great to see that the significance testing added value here. Unfortunately it seems that the authors only implemented these tests for the evaluation against DMS maps, but not for the evaluation against clinical variant sets.

The results for the former also need to be worked into the text better.

For example, the abstract says "The top VEPs are dominated by unsupervised methods including EVE, DeepSequence and ESM-1v, a protein language model that ranked first overall" According to the results, however, ESM-1v was only tied for top rank. As VARITY was tied for second, I suppose one could say that it was outnumbered by unsupervised methods, but perhaps only dominated by one of them?

Similarly, the flow of the results section still states that ESM1-v performed best (followed by a short explanation of how ESM1-v works), only to then state that differences were not significant, which is a bit contradictory. It may be best to lead with the fact that the top performers are statistically indistinguishable. This could also be emphasized in all figures where methods are compared, using brackets and stars to indicate which differences are significant.

Bootstrapping analyses has been added to the **Performance of DMS compared to VEPs against datasets of pathogenic and benign missense variants** and **Benchmarking unsupervised VEPs on large clinical datasets** sections. We have changed the wording and order of several sections in the results and the noted part of the abstract to better reflect the significance of the results.

The statement in the abstract has been changed to: *"Many top VEPs are unsupervised methods…"*

In the **Benchmarking of VEPs using DMS data** section, the details of the bootstrapping are now discussed before the ranking results rather than being introduced after the results are presented. Ranks that do not statistically differ are immediately stated in the text when they appear in this section and in bootstraps in the following sections which were introduced as per this comment.

The results of all bootstrapping analyses have been compiled into one dataset file (EV1) to prevent too many EV tables.

We believe that noting significance in the figures would introduce too much clutter, given so many possible pairwise comparisons, and that the error bars, the EV dataset and text references should be sufficient.

This is a great improvement, although the main text still primarily discusses the old AUROC metric while relegating the AUBPRC result to the supplement.

While we appreciate the potential advantages of the AUBPRC metric, its status as a newly introduced methodology means the caveats and disadvantages of the method are not yet well known. Furthermore, AUROC has been widely studied and the definition well known, and we envision the people who read this paper will be more familiar with ROC as a metric and its interpretation. Thus, we think it is essential to keep the AUROC analysis in the main text. We feel that the manuscript would become overly cluttered by duplicating the analyses in Figs. 3-4 to include both AUROC and AUBPRC in the main text, especially given that the results are very similar.

Therefore, we believe that it is better for the manuscript to leave the main figures and text referring to AUROC, while backing these analyses up with AUBPRC in the EV figures.

This is another case of a new result that needs to be better integrated into the flow of the text. On page 5, the manuscript still describes the original comparison approach which rewards predictors for only reporting on a small fraction of variants instead of penalizing them. The corrected approach is only described much later on page 6. The results of this correction are also not reflected in the main figure 2 and were instead relegated to a supplementary table.

We see this analysis as more in the nature of complementary to the original rather than as a replacement, especially as our analysis is specifically designed to account for the fact that different predictors provide different levels of coverage. Importantly, the altered analysis requires ~70% of the data to be discarded in some cases to cater to nucleotide-level predictors. We believe the original analysis, using as much data as possible, gives a more useful overall result, and this further analysis, while providing very similar results overall, is helpful for identifying those cases where low coverage leads to overperformance (such as in the case of mutationTCN). Again, we think it is better for the overall readability of the manuscript to include this as an EV figure, rather than repeating multiple versions of the analysis in the main text.

"Recalculating the VEP rankings with these data (Table EV9)..." In addition to the need for significance tests noted above, there were no clearly identified ranks in this table.

Re: "The remaining data were used to re-calculate the rank scores (Table EV10).", there were no clearly identified ranks in this table either.

A column with rank number has been added to all ranking tables (Tables EV5 and 6 as well as Datasets EV 2, 3 and 4).

It appears that EV9 and EV10 were originally spreadsheets that were exported to PDF format. Unfortunately that made them hard to read due to columns being split over multiple pages, and some VEP names are cut off. If possible the authors may want to submit the original spreadsheets as supplements instead.

Tables EV8, 9 and 10 have been migrated to EV datasets (2, 3 and 4)

The points made in the context of not screening out benign variants based on allele frequency misses an opportunity to make a point made in both REVEL and VARITY papers, that common benign variants appear not to be representative of rare benign variants. Because the main value of VEPs is to make predictions for rare variants, this would argue that not only should one NOT exclude rare benign variants, there is an argument to include ONLY the subset of benign variants that are rare.

This is an excellent point. We have added text to the **Performance of DMS compared to VEPs against datasets of pathogenic and benign missense variants** section of the Results where the use of the gnomAD dataset is first introduced briefly making this point.

"Since the primary purpose of most VEPs is to assign labels to rare variants that are frequently identified through sequencing, it is potentially more informative to retain these variants in the putatively benign dataset, as has recently been discussed (Wu et al, 2021). Furthermore, as common and rare benign variants may have distinct features (Ioannidis et al, 2016), benchmarking against only common variants is likely to be less reflective of actual clinical utility."

The discussion might note that an alternative 'clean' form of validation for VEPs is the use of prospective or case control cohorts, whereby methods can be ranked according to ability to predict phenotype or case vs. control status.

We have added a paragraph to the Discussion on alternate validation strategies.

"Finding suitable benchmarks to compare VEPs is an ongoing challenge for the field of variant effect prediction, particularly when many of those VEPs are supervised. In addition to DMS datasets, other suitable sources of data that may yield equally bias-free results exist. Prediction performance on case-control disease studies would also not be reliant on existing clinical labels, but would greatly reduce the diversity of variants tested (Wu et al, 2021; McInnes et al, 2019). This approach can also be scaled-up and applied to multiple relevant gene-trait combinations (Kuang et al, 2021)."

Reviewer 3

Reviewers asked about an allele frequency threshold for the benign set from gnomAD and the response of the authors is very surprising here: "Applying an allele frequency threshold was not possible due to the small number of variants remaining". Can the authors please check what is happening here? There should be sufficiently many variants in gnomAD to have a minimum allele frequency threshold for those genes. It does not make sense to use singletons from gnomAD in a benign set. - To cite the authors introduction here: " Pathogenic variants are enriched among the rarest occurring variants in the human population (Wang et al, 2021)". Typically, a frequency threshold of 1 or 5% is used when using "common" variants as benign instead of the biased set of "benign" variants from ClinVar.

First, when running the analyses on a per-protein level, as we need to do in order to test the protein-specific DMS datasets, it is not feasible to apply an allele frequency filter to the gnomAD variants, as this would exclude the large majority of proteins from our analysis. Using an allele frequency cutoff of 1%, none of the 12 human disease-associated proteins with DMS datasets meet our requirement of having at least 10 missense variants with DMS measurements available. Even using a far looser cutoff of 0.01%, only four proteins would be retained. We are absolutely confident that this is correct: most of the genes in this analysis have very few common missense variants in gnomAD. While previous work has commonly used strict allele frequency filters for 'benign' variant datasets, this has typically involved grouping variants across many different protein-coding genes, which is not possible here.

Second, we believe that the exclusion of rare benign variants from this analysis may be detrimental as analysis of rare variants is one of the main tasks that VEPs and DMS are useful for. Rare benign variants may also have distinct properties from common ones, resulting in a poor benchmark if only the common variants are retained. We also note that Reviewer 1 supports our use of rare putatively benign variants from gnomAD stating *"Because the main value of VEPs is to make predictions for rare variants, this would argue that not only should one NOT exclude rare benign variants, there is an argument to include ONLY the subset of benign variants that are rare."* As described above, we have expanded upon the point in the text.

Along those lines, it should be possible to match the number of common and pathogenic variants in this analysis on a gene level, rather than some overall correction for balanced AUROCs. It is important to represent genes with the same number of pathogenic and benign variants. Otherwise, you are starting to test whether scores identify genes with many (known) pathogenic variants, i.e., whether they work on a gene level and not a variant level. The intuition with which the use of

AUROCs is motivated in the manuscript (i.e., that it does not matter if certain genes are better covered with pathogenic variants) is therefore not correct.

In addition to the more traditional AUROC measure, we have also used AUBPRC in this study. AUBPRC is a derivative of the precision-recall curve, rather than the ROC curve, so like precision-recall, it takes into account the predictive performance on true positives both against all positives and all predicted positives. Unlike precision-recall, it remains comparable when the sample size changes. We believe our inclusion of AUBPRC-based results to sufficiently back-up any potential weaknesses in AUROC as a metric, but note that most give very similar results. Moreover, the high correlation of rankings between independent DMS and clinical variant benchmarks strongly supports the validity of our approach.

Considering the prominent discussion about assigning Eigen as a supervised method, maybe it is worthwhile to also discuss CADD, DANN, PrimateAI and probably some others, which also trained on labeled data (thus are in the supervised group), but not pathogenic/benign labels.

The reason Eigen is singled out was because it is an exception to our simple classification system, and we wanted to clarify this. It is based on an unsupervised learning approach, but because it includes the supervised predictor PolyPhen-2 as a feature, it does potentially suffer from data circularity concerns. CADD, DANN and PrimateAI make use of simulated variants, and may perhaps be less biased than other supervised VEPs, but model optimisation was still performed using a labelled dataset, and thus concerns about circularity still remain. Importantly, none of these predictors ranked highly in either benchmark, so concerns about whether circularity is inflating their apparent performance have little bearing on our overall findings.

Typos:

- "\Introduction"

- "which makes comparisons [of] different proteins difficult"

- "from 985 protein<s>."

We have corrected all of the above typos.

2nd Jun 2023

Manuscript number: MSB-2022-11474RR
Title: Updated benchmarking of variant effect predictors using deep mutational scanning

Dear Dr. Marsh,

Thank you again for sending us your revised manuscript. We are now satisfied with the modifications made and I am pleased to inform you that your paper has been accepted for publication.

*** PLEASE NOTE *** As part of the EMBO Publications transparent editorial process initiative (see our Editorial at https://dx.doi.org/10.1038/msb.2010.72), Molecular Systems Biology publishes online a Review Process File with each accepted manuscripts. This file will be published in conjunction with your paper and will include the anonymous referee reports, your point- by-point response and all pertinent correspondence relating to the manuscript. If you do NOT want this File to be published, please inform the editorial office at msb@embo.org within 14 days upon receipt of the present letter.

Should you be planning a Press Release on your article, please get in contact with msb@wiley.com as early as possible, in order to coordinate publication and release dates.

LICENSE AND PAYMENT:
All articles published in Molecular Systems Biology are fully open access: immediately and freely available to read, download and share.

Molecular Systems Biology charges an article processing charge (APC) to cover the publication costs. You, as the corresponding author for this manuscript, should have already received a quote with the article processing fee separately. Please let us know in case this quote has not been received.

Once your article is at Wiley for editorial production you will receive an email from Wiley's Author Services system, which will ask you to log in and will present you with the publication license form for completion. Within the same system the publication fee can be paid by credit card, an invoice or pro forma can be requested.

Payment of the publication charge and the signed Open Access Agreement form must be received before the article can be published online.

Molecular Systems Biology articles are published under the Creative Commons licence CC BY, which facilitates the sharing of scientific information by reducing legal barriers, while mandating attribution of the source in accordance to standard scholarly practice.

Proofs will be forwarded to you within the next 2-3 weeks.

Thank you very much for submitting your work to Molecular Systems Biology.

Kind regards,

Maria

Maria Polychronidou, PhD
Senior Editor
Molecular Systems Biology

EMBO Press Author Checklist

Corresponding Author Name: Dr Joseph Marsh
Journal Submitted to: Molecular Systems Biology
Manuscript Number: MSB-2022-11474

USEFUL LINKS FOR COMPLETING THIS FORM
The EMBO Journal - Author Guidelines
EMBO Reports - Author Guidelines
Molecular Systems Biology - Author Guidelines
EMBO Molecular Medicine - Author Guidelines

Reporting Checklist for Life Science Articles (updated January
This checklist is adapted from Materials Design Analysis Reporting (MDAR) Checklist for Authors. MDAR establishes a minimum set of requirements in transparent reporting in the life sciences (see Statement of Task: 10.31222/osf.io/9sm4x). Please follow the journal's guidelines in preparing your
Please note that a copy of this checklist will be published alongside your article.

Abridged guidelines for figures
1. Data
The data shown in figures should satisfy the following conditions:
- the data were obtained and processed according to the field's best practice and are presented to reflect the results of the experiments in an accurate and unbiased manner.
- ideally, figure panels should include only measurements that are directly comparable to each other and obtained with the same assay.
- plots include clearly labeled error bars for independent experiments and sample sizes. Unless justified, error bars should not be shown for technical
- if n<5, the individual data points from each experiment should be plotted. Any statistical test employed should be justified.
- Source Data should be included to report the data underlying figures according to the guidelines set out in the authorship guidelines on Data

2. Captions
Each figure caption should contain the following information, for each panel where they are relevant:
- a specification of the experimental system investigated (eg cell line, species name).
- the assay(s) and method(s) used to carry out the reported observations and measurements.
- an explicit mention of the biological and chemical entity(ies) that are being measured.
- an explicit mention of the biological and chemical entity(ies) that are altered/varied/perturbed in a controlled manner.
- the exact sample size (n) for each experimental group/condition, given as a number, not a range;
- a description of the sample collection allowing the reader to understand whether the samples represent technical or biological replicates (including how many animals, litters, cultures, etc.).
- a statement of how many times the experiment shown was independently replicated in the laboratory.
- definitions of statistical methods and measures:
  - common tests, such as t-test (please specify whether paired vs. unpaired), simple $\chi 2$ tests, Wilcoxon and Mann-Whitney tests, can be unambiguously identified by name only, but more complex techniques should be described in the methods section;
  - are tests one-sided or two-sided?
  - are there adjustments for multiple comparisons?
  - exact statistical test results, e.g., P values = x but not P values < x;
  - definition of 'center values' as median or average;
  - definition of error bars as s.d. or s.e.m.

Please complete ALL of the questions below.
Select "Not Applicable" only when the requested information is not relevant for your study.

Materials

Newly Created Materials	Information included in the manuscript?	In which section is the information available? (Reagents and Tools Table, Materials and Methods, Figures, Data Availability Section)
New materials and reagents need to be available; do any restrictions apply?	Not Applicable	

Antibodies	Information included in the manuscript?	In which section is the information available? (Reagents and Tools Table, Materials and Methods, Figures, Data Availability Section)
For **antibodies** provide the following information:<br>- Commercial antibodies: RRID (if possible) or supplier name, catalogue number and or/clone number<br>- Non-commercial: RRID or citation	Not Applicable	

DNA and RNA sequences	Information included in the manuscript?	In which section is the information available? (Reagents and Tools Table, Materials and Methods, Figures, Data Availability Section)
**Short novel DNA or RNA including primers, probes**: provide the sequences.	Not Applicable	

Cell materials	Information included in the manuscript?	In which section is the information available? (Reagents and Tools Table, Materials and Methods, Figures, Data Availability Section)
**Cell lines:** Provide species information, strain. Provide accession number in repository **OR** supplier name, catalog number, clone number, and/**OR** RRID.	Not Applicable	
**Primary cultures:** Provide species, strain, sex of origin, genetic modification status.	Not Applicable	
Report if the cell lines were recently **authenticated** (e.g., by STR profiling) and tested for mycoplasma contamination.	Not Applicable	

Experimental animals	Information included in the manuscript?	In which section is the information available? (Reagents and Tools Table, Materials and Methods, Figures, Data Availability Section)
**Laboratory animals or Model organisms:** Provide species, strain, sex, age, genetic modification status. Provide accession number in repository **OR** supplier name, catalog number, clone number, **OR** RRID.	Not Applicable	
**Animal observed in or captured from the field:** Provide species, sex, and age where possible.	Not Applicable	
Please detail **housing and husbandry conditions**.	Not Applicable	

Plants and microbes	Information included in the manuscript?	In which section is the information available? (Reagents and Tools Table, Materials and Methods, Figures, Data Availability Section)
**Plants:** provide species and strain, ecotype and cultivar where relevant, unique accession number if available, and source (including location for collected wild specimens).	Not Applicable	
**Microbes:** provide species and strain, unique accession number if available, and source.	Not Applicable	

Human research participants	Information included in the manuscript?	In which section is the information available? (Reagents and Tools Table, Materials and Methods, Figures, Data Availability Section)
If collected and within the bounds of privacy constraints report on age, sex and gender or ethnicity for all study participants.	Not Applicable	

Core facilities	Information included in the manuscript?	In which section is the information available? (Reagents and Tools Table, Materials and Methods, Figures, Data Availability Section)
If your work benefited from core facilities, was their service mentioned in the acknowledgments section?	Not Applicable	

Design

Study protocol	Information included in the manuscript?	In which section is the information available? (Reagents and Tools Table, Materials and Methods, Figures, Data Availability Section)
If study protocol has been **pre-registered, provide DOI in the manuscript**. For clinical trials, provide the trial registration number **OR** cite DOI.	Not Applicable	
Report the **clinical trial registration number** (at ClinicalTrials.gov or equivalent), where applicable.	Not Applicable	

Laboratory protocol	Information included in the manuscript?	In which section is the information available? (Reagents and Tools Table, Materials and Methods, Figures, Data Availability Section)
Provide DOI OR other citation details if **external detailed step-by-step protocols** are available.	Not Applicable	

Experimental study design and statistics	Information included in the manuscript?	In which section is the information available? (Reagents and Tools Table, Materials and Methods, Figures, Data Availability Section)
Include a statement about **sample size** estimate even if no statistical methods were used.	Not Applicable	
Were any steps taken to minimize the effects of subjective bias when allocating animals/samples to treatment (e.g. **randomization procedure**)? If yes, have they been described?	Not Applicable	
Include a statement about **blinding** even if no blinding was done.	Not Applicable	
Describe **inclusion/exclusion criteria** if samples or animals were excluded from the analysis. Were the criteria pre-established?<br><br>If sample or data points were omitted from analysis, report if this was due to attrition or intentional exclusion and provide justification.	Not Applicable	
For every figure, are **statistical tests** justified as appropriate? Do the data meet the assumptions of the tests (e.g., normal distribution)? Describe any methods used to assess it. Is there an estimate of variation within each group of data? Is the variance similar between the groups that are being statistically compared?	Not Applicable	

Sample definition and in-laboratory replication	Information included in the manuscript?	In which section is the information available? (Reagents and Tools Table, Materials and Methods, Figures, Data Availability Section)
In the figure legends: state number of times the experiment was **replicated** in laboratory.	Not Applicable	
In the figure legends: define whether data describe **technical or biological replicates**.	Not Applicable	

Ethics

Ethics	Information included in the manuscript?	In which section is the information available? (Reagents and Tools Table, Materials and Methods, Figures, Data Availability Section)
Studies involving **human participants**: State details of **authority granting ethics approval** (IRB or equivalent committee(s), provide reference number for approval.	Not Applicable	
Studies involving **human participants**: Include a statement confirming that **informed consent** was obtained from all subjects and that the experiments conformed to the principles set out in the WMA Declaration of Helsinki and the Department of Health and Human Services Belmont Report.	Not Applicable	
Studies involving **human participants:** For publication of **patient photos**, include a statement confirming that consent to publish was obtained.	Not Applicable	
Studies involving experimental **animals**: State details of **authority granting ethics approval** (IRB or equivalent committee(s), provide reference number for approval. Include a statement of compliance with ethical regulations.	Not Applicable	
Studies involving **specimen and field samples:** State if relevant **permits** obtained, provide details of authority approving study; if none were required, explain why.	Not Applicable	

Dual Use Research of Concern (DURC)	Information included in the manuscript?	In which section is the information available? (Reagents and Tools Table, Materials and Methods, Figures, Data Availability Section)
Could your study fall under dual use research restrictions? Please check biosecurity documents and list of **select agents and toxins** (CDC): https://www.selectagents.gov/sat/list.htm	Not Applicable	
If you used a select agent, is the security level of the lab appropriate and reported in the manuscript?	Not Applicable	
If a study is subject to dual use research of concern regulations, is the name of the **authority granting approval and reference number** for the regulatory approval provided in the manuscript?	Not Applicable	

Reporting

The MDAR framework recommends adoption of discipline-specific guidelines, established and endorsed through community initiatives. Journals have their own policy about requiring specific guidelines and recommendations to complement MDAR.

Adherence to community standards	Information included in the manuscript?	In which section is the information available? (Reagents and Tools Table, Materials and Methods, Figures, Data Availability Section)
State if relevant guidelines or checklists (e.g., **ICMJE, MIBBI, ARRIVE, PRISMA**) have been followed or provided.	Not Applicable	
For **tumor marker prognostic studies,** we recommend that you follow the **REMARK** reporting guidelines (see link list at top right). See author guidelines, under 'Reporting Guidelines'. Please confirm you have followed these guidelines.	Not Applicable	
For **phase II and III randomized controlled trials**, please refer to the **CONSORT** flow diagram (see link list at top right) and submit the CONSORT checklist (see link list at top right) with your submission. See author guidelines, under 'Reporting Guidelines'. Please confirm you have submitted this list.	Not Applicable	

Data Availability

Data availability	Information included in the manuscript?	In which section is the information available? (Reagents and Tools Table, Materials and Methods, Figures, Data Availability Section)
Have **primary datasets** been deposited according to the journal's guidelines (see 'Data Deposition' section) and the respective accession numbers provided in the Data Availability Section?	Yes	Data availability
Were **human clinical and genomic datasets** deposited in a public access-controlled repository in accordance to ethical obligations to the patients and to the applicable consent agreement?	Not Applicable	
Are **computational models** that are central and integral to a study available without restrictions in a machine-readable form? Were the relevant accession numbers or links provided?	Not Applicable	
If publicly available data were reused, provide the respective **data citations in the reference list**.	Not Applicable	
